# Improving eye-drop administration skills of patients – A multicenter parallel-group cluster-randomized controlled trial

**DOI:** 10.1371/journal.pone.0212007

**Published:** 2019-02-21

**Authors:** Anette Lampert, Thomas Bruckner, Walter E. Haefeli, Hanna M. Seidling

**Affiliations:** 1 Department of Clinical Pharmacology and Pharmacoepidemiology, Heidelberg University, Heidelberg, Germany; 2 Cooperation Unit Clinical Pharmacy, Heidelberg University, Heidelberg, Germany; 3 Institute of Medical Biometry and Informatics, Heidelberg University, Heidelberg, Germany; Universita degli Studi di Firenze, ITALY

## Abstract

**Background:**

Eye-drop administration errors occur in the majority of patients and increase the risk for treatment failure or systemic adverse events. While lacking knowledge is the principal error cause, most patients overestimate their skills and are unaware of often substantial knowledge gaps. Therefore, the impact of including motivational patient education on long-term eye-drop administration skills of patients was investigated.

**Methods:**

This is a cluster-randomized controlled trial in German community pharmacies. Patient education in both groups comprised observation of the patient during eye-drop administration to identify individual errors, pharmaceutical counseling, and teach-back evaluation of the training. In the intervention group, motivational communication techniques were included to increase error awareness and readiness for patient education. In addition, intervention patients were trained on repeated errors until administration was performed correctly. In contrast, patients in the control group only received feedback on erroneous administration steps without another assessment and reinforced training.

**Results:**

In total, 152 adult patients were eligible to the study and 91 patients (intervention group N = 46) agreed to participate in a 1-month, 6-month, and 12-month follow-up. Patient education significantly increased the proportion of patients correctly administering eye-drops from 6% (7 out of 56 intervention patients, 1 out of 82 control patients) at baseline to 35% (12 out of 30 intervention patients, 12 out of 39 control patients, p ≤ 0.001) at the 1-month follow-up, and 64% (11 out of 15 intervention patients, 17 out of 29 control patients, p ≤ 0.001) at the 6-month follow-up irrespective of group allocation. In some patients previously resolved errors recurred during follow-up visits. This emphasizes the need for periodical reevaluation of patient administration skills and the provision of prevention strategies besides education.

**Conclusion:**

Patient education that included demonstration of administration skills and verbal and written counseling on observed errors improved eye-drop administration skills irrespective of the communication technique applied. Whereof, high drop-out rates limited the power to detect a difference between groups. In particular, periodic demonstration of administration skills seemed important for sustainable improvement of administration skills. However, further error prevention strategies such as additional education materials or support by a caregiver may be necessary in some patients.

## Introduction

The eye-drop administration process comprises multiple action steps with various opportunities for error that can cause treatment failure or adverse drug events.[1–7] However, the majority of patients do not receive education on correct eye drop use.[8, 9]Up to 9 out of 10 glaucoma patients are unable to correctly instill their eye-drops[2] even with long-term eye-drop use.[3, 8] In addition, patients continue to overestimate their administration skills.[3, 8] Most often, lacking knowledge is the principal cause of error, making patient education a promising strategy to prevent knowledge-based administration errors.[10] However, lacking knowledge is often accompanied by unawareness of potential pitfalls.[2, 8, 11] Subsequently, patients underestimate possible consequences of poor eye-drop administration technique, which may negatively influence their decision to participate in training sessions.[2, 3, 8, 12] In addition, eye-drop administration skills are often limited by insufficient physical capabilities such as decreased finger strength and vision.[1, 3] Typically, multiple interdependent factors co-create administration errors and, thus, observation of patient administration skills and inclusion of the patient perspective may facilitate identification of individual barriers.[13] Therefore, we investigated a structured patient education program that included a joint exploration of individual administration problems and provision of tailored counseling in German community pharmacies.

## Materials and methods

The study was part of a patient education program to improve administration of several error-prone dosage forms. It was approved by the Ethics Committee of the Medical Faculty of Heidelberg University (S-492/2014) November 26^th^, 2014 and registered in the German Clinical Trials Register (DRKS00008941). The trial protocol can be accessed as supporting information. The study was registered after recruiting of the community pharmacies and inclusion of pharmacy staff but before the first patient was included to the study. The authors confirm that all ongoing and related trials for this intervention are registered.

### Study design and randomization

In April 2015, German community pharmacies were invited by the Chamber of Pharmacists Baden-Wuerttemberg to participate in a multicenter, parallel-group, cluster-randomized, controlled trial to improve counseling on correct medication administration. Cluster-randomization into intervention or control group was performed on pharmacy level to prevent contamination between intervention and control group. Computer-generated randomization was performed using www.randomization.com. AL allocated community pharmacies to intervention or control group according to sequence of agreement with the head of the pharmacy. Pharmacy staff were not blinded to group allocation.

### Study procedures

Pharmacy staff in both groups were informed about the study procedure and received training on correct medication administration in an approximately 60-min seminar. In the intervention group, the seminar was expanded to approximately 120 min to additionally teach suitable communication techniques including the spirit of motivational interviewing.[14] There were no regular refresher trainings implemented. However, pharmacies were regularly visited to clarify questions and to re-train communication techniques. Motivational interviewing is a counseling technique that focuses on listening to the patient in order to understand and explore patient motivation.[14] Therefore, the communication training in the intervention group particularly emphasized listening to the patient’s perspective on medication administration and taking into account that patients are probably not aware of errors. In the control group, pharmacy staff were not explicitly trained on communication techniques that target poor motivation of patients. During their daily routine, pharmacy staff screened customers who obtained eye-drops for eligibility Patients were asked whether they used eye-drops for the first time or whether they used this dosage form regularly. Patients, who regularly used eye-drops were then asked to rate their administration skills as “very good“, „good“, “sometimes good, sometimes poor”, “less good”, “not good”, or “I don’t know” (S1 Fig). Subsequently, pharmacy staff assessed individual administration skills of regular eye-drop users by observing the patient administering placebo eye-drops and ticking off correctly performed administration steps on a pre-defined checklist. Eye-drop administration was considered correct when patients carried out all clinically relevant administration steps that were defined according to published eye-drop administration errors, reported adverse events, and manufacturer recommendations (Table 1).

**Table 1:** Clinically relevant steps in the eye-drop administration process.

Administration steps	Clinical relevance of incorrect execution	Principal cause of error
Hand washing*	Manufacturer recommendation	Lacking knowledge
Instillation of a single drop	Systemic adverse events resulting from instillation of multiple drops and absorption through the lacrimal drainage system[2, 3, 10, 15]	Lacking skills
Instillation into the conjunctival sac	The drop misses the eye resulting in local side effects and waste of eye-drops, which increases treatment costs[2, 3, 12]	Lacking skills
Eyelid closure for approximately one minute	Systemic adverse events because eyelid closure is omitted and ocular drugs become systemically available through the lacrimal drainage system[2, 6, 7, 15, 16]	Lacking knowledge
Concomitant nasolacrimal occlusion	Systemic adverse events because nasolacrimal duct is not occluded and ocular drugs become systemically available through the lacrimal drainage system[2, 6, 7, 15, 16]	Lacking knowledge
Avoid touching the dropper tip	Trauma, infection, ulceration, and visual loss resulting from contamination of the bottle tip by touching the ocular surface or eyelid[2–5, 10, 12, 17, 18]	Lacking skills

*Mentioning this step was sufficient for rating as correct.

Patients who correctly administered their eye-drops were not included in the study but were asked whether the completed checklist could be kept anonymously to determine the total number of patients assessed for administration errors. Patients with at least one missing or incorrect administration step were trained according to group allocation. Patients who used eye-drops for the first time were immediately counseled. In both groups, pharmacists used information leaflets to structure the training session. Counseling in the intervention group additionally included aspects of the previously performed communication training that aimed at increasing the patients’ awareness of the importance of correct eye-drop administration and, thus, motivation for a permanent change of their eye-drop administration routine. Subsequently, teaching success was evaluated by observing patients administering placebo eye-drops. If an error re-occurred, patients in the intervention group were trained on individual errors with repeated observations until administration was performed correctly, whereas patients in the control group only received feedback on erroneous administration steps without another assessment. Afterwards, pharmacy staff informed patients about the study and invited them for follow-up visits after one month, six months, and twelve months to re-evaluate administration skills. At follow-up visits, pharmacy staff assessed administration skills of patients and provided counseling when necessary, followed by a teach-back evaluation. For evaluation of practicability, pharmacy staff in both groups started recording the time as soon as an eligible candidate for the study was identified until patient education was completed. The time difference allowed estimation of the net duration of the patient education including the baseline assessment and evaluation of teaching success.

### Participants: Recruitment and eligibility

Community pharmacies with premises for confidential consultation were included after pharmacy staff gave their written informed consent. Adult customers (age ≥ 18 years) who obtained eye-drops were cognitively and physically capable to participate and self-administered eye-drops were considered eligible for the study. In case a caregiver administered the eye-drops for the patient, the caregiver would be eligible for study participation and trained on correct administration technique. Study information was given to the patients after the first training session in order to simulate usual counseling procedures during drug dispensing without study-specific interruptions, such as signing informed consent. If participants gave their written informed consent, a pseudonymous identifier was allocated to the previously completed checklist to allow matching of follow-up visits with the baseline assessment. This procedure was approved by the Ethics Committee of the Medical Faculty of Heidelberg University (S-492/2014). All patients included to the follow-up gave their written informed consent. Pharmacy staff recruited patients from May 2015 until August 2016. The follow-up continued until September 2017.

### Study materials

Placebo eye-drops were provided by the pharmacy of Heidelberg University Hospital in a polyethylene dropper bottle containing 3 ml sterile 0.9% saline without preservatives. In both groups, pharmacy staff used information leaflets, which contained recommendations for correct eye-drop use and were previously validated for comprehensibility [19].

### Primary outcome

The primary outcome measure was the comparison of the number of patients who correctly performed eye-drop administrations between intervention and control group before counseling after six months.

### Secondary outcomes

Secondary outcomes were (i) the number of patients who correctly administered their eye-drops before and after patient education at the first encounter as a measure for the immediate impact of patient education on administration skills, (ii) the type of administration errors at baseline, and (iii) the number of patients who correctly administered their eye-drops after one month and six months compared to baseline as a measure for the sustainability of patient education. In addition, (iv) the patients’ perspective on their administration skills was obtained by self-evaluation of drug administration before patient education and compared to their demonstrated skills.

### Sample size calculation

Sample size calculation was based on a randomized trial that compared a teach-to-goal intervention for the correct use of a metered-dose inhaler with a brief intervention comparable to routine training.[20] Assuming a rate of p1 = 32% in group A and p2 = 52% in group B, n = 95 participants per group are needed to evaluate this difference of 20% when applying a chi-squared test (alpha = 5%, beta = 20%). Considering a drop-out rate of 15% after six months, a cluster size of 10 community pharmacies, and an intra-cluster correlation coefficient (icc) of 0.02, a number of N = 133 participants per group was required for a significant difference using chi-squared test (unpaired, two-sided, α = 0.05; β = 0.8).

During conduct of the study it became evident that participating pharmacies had difficulties with recruiting patients. Therefore, after approximately one year, the study was stopped and included patients were analyzed.

### Statistical analysis

Comparisons between groups were calculated using a chi-squared test. Before–after comparisons were calculated using a McNemar test. Statistical analysis was performed using IBM SPSS Statistics Version 24. The 12-month follow-up was not analyzed because of low numbers of patients available for the 12-month follow-up at the time of the end of the study. The level of significance was set at p ≤ 0.05. Because of the exploratory character of the study, p-values were interpreted accordingly.

## Results

**Fig 1:**
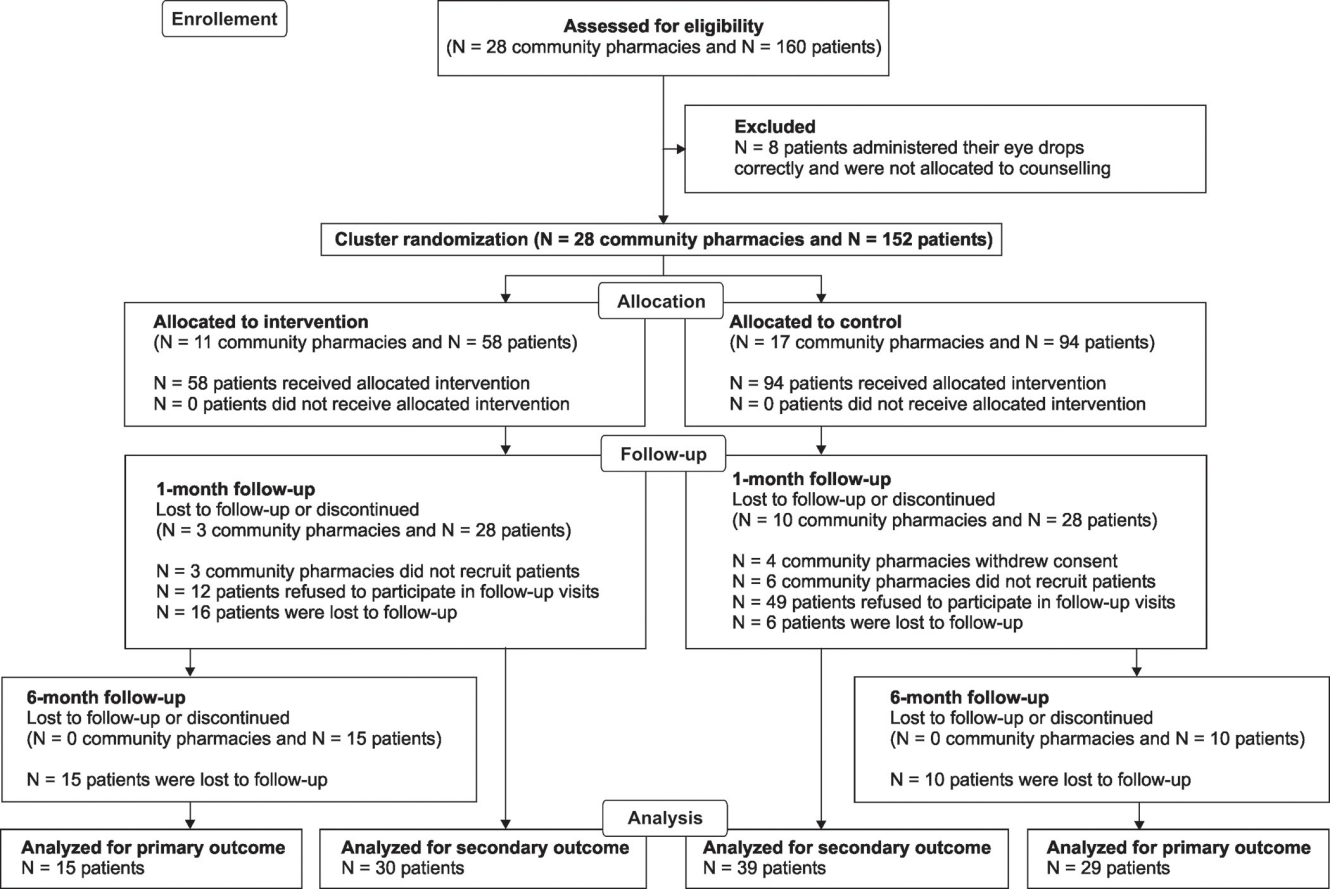
Flow diagram of the study.

A total of 28 German community pharmacies were randomly allocated to intervention (N = 11) or control group (N = 17) (Fig 1). Four community pharmacies in the control group withdrew consent prior to including patients into the study because of time constraints. In the intervention group eight community pharmacies and in the control group seven community pharmacies included patients into the study. From May 2015 until August 2016, pharmacy staff screened 160 patients for eye-drop administration errors. All patients self-administered their eye-drops.

In the intervention group, 56 regular eye-drop users were assessed for administration errors and 9 patients were immediately taught about correct administration because they used eye-drops for the first time. In the control group, 82 patients used eye-drops regularly and were eligible for baseline assessment, while 13 first-time eye-drop users were counseled. Seven patients in the intervention group and one patient in the control group administered their eye-drops correctly at baseline and, thus, received no counseling and were not included to follow-up visits. Hence, 152 patients were eligible for the study and 91 patients (60%) agreed to participate in follow-up visits: The proportion of patients agreeing to participate was 79% (46 out of 58 eligible patients) in the intervention group and 48% (45 out of 94) in the control group (p ≤ 0.001). Most of the included patients (80%) reported not having received previous education on correct eye-drop administration (Table 2). In the control group twice as many patients previously received training on correct eye-drop administration compared to intervention patients (p = 0.01) without specifying the content of the training. The duration of patient education was comparable in both groups (S1 Table).

**Table 2:** Demographic data of study participants invited to follow-up visits.

	Intervention (N = 46)	Control (N = 45)	p-value
Female sex N (%)	29 (63 %)	25 (56 %)	0.31*
Age (mean ± SD)	61.3 ± 15.4 years	60.5 ± 16.7 years	0.96°
Native language N (%)			1*
German	42 (91 %)	41 (91 %)	
other	4 (9 %)	4 (9 %)	
Number of regularly administered drugs N (median, IQR)	2 (1–4)	3 (1–5)	0.55°
Number of patients who used eye-drops for			0.98*
less than one year N (%)	22 (48 %)	19 (42 %)	
more than one year N (%)	24 (52 %)	26 (58 %)	
Previous education on eye-drop administration N (%)	6 (13 %)	12 (27 %)	0.01*

IQR = Interquartile range, SD = standard deviation

*p-values were calculated using a chi-squared test.

°p-values were calculated using a Mann-Whitney U test.

**Fig 2:**
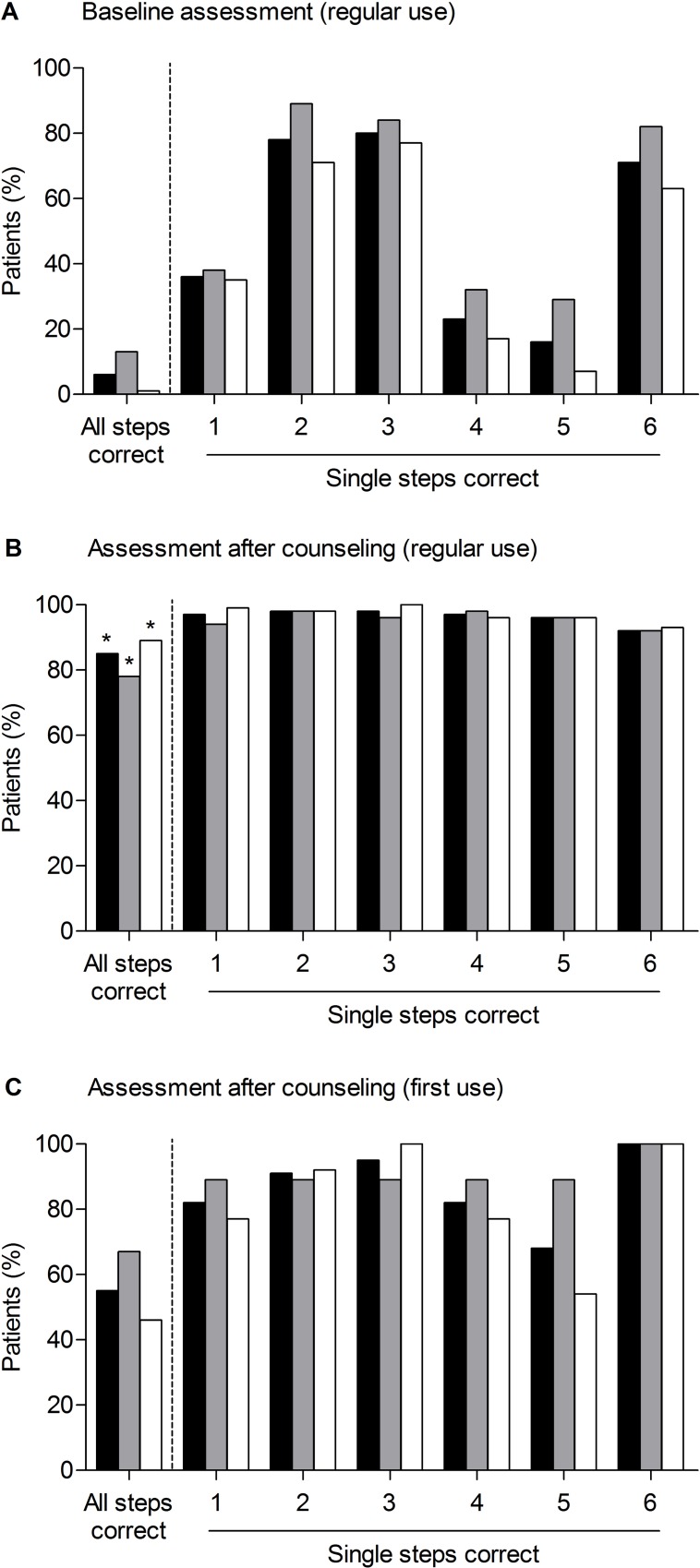
Patients who correctly administered their eye-drops before and after counseling. Correctly performed eye-drop administration at baseline of all patients (black), intervention group (grey), and control group (white) who regularly used eye-drops (A). Correctly performed eye-drop administration after counseling of all patients (black), intervention group (grey), and control group (white) who regularly administer eye-drops (B). Seven patients in the intervention group and one patient in the control group correctly administered eye-drops at baseline and, thus, received no counseling (C). Correctly performed eye-drop administration after counseling of all patients (black), intervention group (grey), and control group (white) using eye-drops for the first time (bottom). The eye-drop administration process comprised six steps: 1 = hand washing, 2 = instilling a single drop, 3 = instillation into the conjunctival sac, 4 = eyelid closure for approximately one minute, 5 = concomitant nasolacrimal occlusion, and 6 = dropper tip was not touched. *significant compared to baseline (p ≤ 0.001).

**Fig 3:**
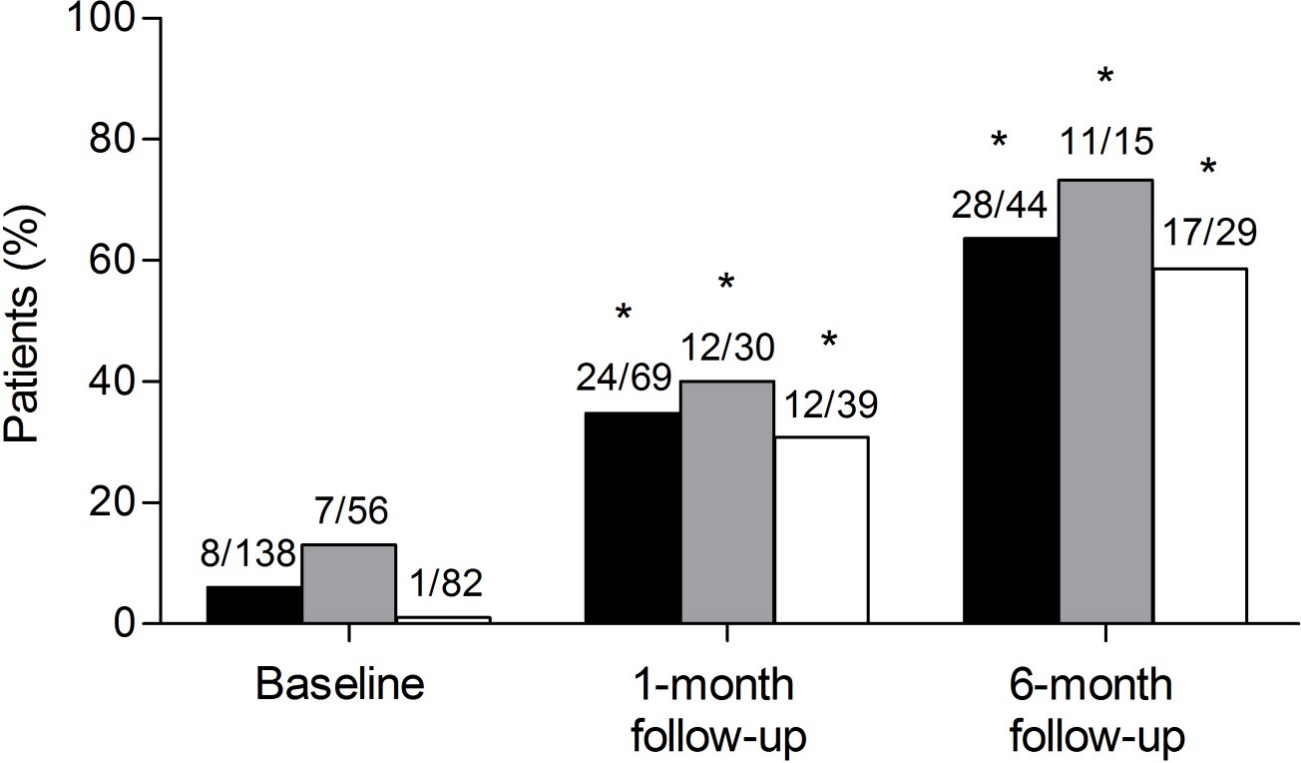
Patients who correctly administered their eye-drops at baseline and follow-ups. Number of patients who correctly administered eye-drops prior to counseling at baseline, 1-month follow-up, and 6-month follow-up in relation to the total number of patients (black), all patients in the intervention group (grey), or in the control group (white) who were assessed for eye-drop administration errors. *significant compared to baseline (p ≤ 0.001).

The majority of patients used eye-drops regularly (S2 Table) and rated their administration skills as “very good” (20%, 28 out of 138) or “good” (48%, 66 out of 138) (S2 Fig). Patient education significantly increased the proportion of patients with correct administration skills by 79% (6%, N = 8 to 85%, N = 110; p ≤ 0.001) during the first encounter (Fig 2A and 2B). The greatest improvement was achieved in knowledge-based errors, i.e. omission of hand washing, eyelid closure, and nasolacrimal occlusion, which were the most common errors during baseline assessment. More than half of the first-time users could administer eye-drops correctly after counseling irrespective of group allocation (Fig 2C). Compared to baseline, patient education sustainably improved eye-drop administration skills after one month (35% patients correctly administered eye-drops, p ≤ 0.001) and after six months (64%, p ≤ 0.001) (Fig 3). At the 6-month follow-up, the skills assessment before counseling revealed no difference between intervention (73%, 11 out of 15) and control group (59%, 17 out of 29; p = 0.5) in the number of patients with correct eye-drop administration and again most patients rated their eye-drop administration skills as “very good” (intervention group 67%, N = 10; control group 41%, N = 12) or “good” (intervention group 27%, N = 4; control group 55%, N = 16). After 12 months four intervention patients and one control patient came to the community pharmacy and correctly administered their eye-drops correctly.

Overall, 15 intervention and 29 control patients attended both follow-up visits (Fig 1). Errors were permanently resolved in six patients (40%) in the intervention group (S3 Fig, Patients 1–6) and eight patients (28%) in the control group (S4 Fig, Patients 1–8). In two control patients the same errors persisted throughout the follow-up visits and could not be resolved through education (S4 Fig, Patients 28 and 29). In eight intervention patients (53%) (S3 Fig, Patients 8–14) and sixteen control patients (55%) (S4 Fig, Patients 12–27) errors that seemed to be resolved through patient education recurred during follow-up visits.

## Discussion

Correct medication administration is crucial to effective drug treatment. The majority of our patients could not administer their eye-drops correctly although most of them used their medication regularly. Knowledge-based errors such as omission of eyelid closure combined with nasolacrimal occlusion seemed unknown or forgotten by most patients. However, both steps crucially influence treatment outcome by increasing ocular drug availability [16, 21, 22] and reducing systemic adverse events [6, 7, 15, 16]. Patient education significantly improved these knowledge-based errors, irrespective of the communication technique applied. Although motivational interviewing was not superior in improving administration skills, the communication technique may have stimulated the willingness of patients to participate in patient education. Compared to the intervention group, pharmacy staff in the control group had difficulties to motivate their patients for continuous re-evaluation. It seemed that patients in the intervention group appreciated the offer for re-evaluations of administration skills. In both groups, the patients received comprehensible written information material [19] that probably already facilitated sustainable gain of knowledge [23, 24]. However, patient education even improved skill-based errors, such as instillation of multiple drops, misdirection of the eye, and contamination of the bottle tip. The demonstration of eye-drop administration in front of a healthcare professional and subsequent correction of errors by re-training or verbal counseling through pharmacy staff probably contributed most to improvement of administration skills. Particularly iterative training of observed errors in the intervention group might have contributed to the larger proportion of patients whose errors seemed permanently resolved at follow-up. Demonstration of eye-drop administration is rarely included in patient education [9] although the teach-back technique, which means that a patient explains or demonstrates a skill just learned back to the instructor, is considered an effective method for improving medication administration skills.[25, 26] In most studies that showed the effectiveness of patient education on improved administration skills, iterative evaluations of the patient’s capability to administer drugs correctly were lacking.[9, 27] However, every second patient, who attended both follow-up visits, repeated an error that seemed already resolved through the baseline training session. Mostly, omission of eyelid closure and nasolacrimal occlusion reoccurred, probably because both steps were new to most patients, thus requiring long-term changes of their administration routine and repeated education. Therefore, regular reevaluation of administration skills and patient education seems necessary to continuously assure and improve administration skills and to prevent relapse into previous administration patterns.[2, 28–30] In addition, errors persisted in some patients throughout the follow-up visits probably requiring additional education materials (e.g., videos[27]) or further error prevention strategies that go beyond patient education (e.g., helping aids[31] or support by a caregiver[32]). Therefore, further studies should identify patient characteristics that predict the capability to improve eye-drop administration skills through education to allow for more evidence-based recommendations on patient education for correct eye-drop administration. Irrespective of their actual competence, most patients rated their administration skills as “very good” or “good” prior to counseling confirming that patients frequently overestimate their administration skills.[3, 8, 12] However, patients were probably unaware of all relevant administration steps and considered their skills as appropriate as long as the drop reached the eye. Therefore, the assessment of administration skills should by objectified by direct observation of eye-drop administration.[9]

This study has several limitations. The results are limited by the small number of patients, which impaired the evaluation of the impact of a motivational communication technique on patient administration skills. High drop-out rates were possibly promoted by the long follow-up of six months, which was chosen to evaluate whether a time-consuming intervention would pay off in the long run because the training effect would last longer. However, studies should determine the appropriate interval to re-evaluate administration skills and need for education. In addition, cluster-randomization possibly introduced a bias for example when patients in one pharmacy have previously received patient education while patients other in another pharmacy have not [33]. For example, more included control patients previously received training on eye-drop administration compared to intervention patients, which could have biased results through a cluster-effect. The communication techniques adopted from motivational interviewing may have been insufficient to cause a difference between control and intervention group. Motivational interviewing is a rather complex intervention, which is difficult to implement in ad-hoc counseling situations such as in community pharmacies.[14] The used placebo dosage forms simulated usual characteristics of eye-drop preparations, which may have differed from patients’ individual medicines. For example, a more rigid bottle would impair drop removal compared to the used more flexible polyethylene bottle [34]. In addition, we did not assess the indication why the patients were using eye-drops. However, disease burden may have an impact on success of motivational interviewing and should be investigated in further trials. The study may have been exposed to selection bias because pharmacy staff that are more motivated would participate in the study as would highly-motivated patients. In addition, a Hawthorne effect (i.e., modification of behavior due to observation) may have influenced the assessment of patient administration skills in the pharmacy, which would probably result in an overestimation of teaching success. The dropout rates could have overestimated results at follow-up visits, because patients with problems during administration may not have revisited the pharmacy.

In conclusion, in both groups patient education improved eye-drop administration but high drop-out rates limited the power to detect differences between groups. The joint exploration of errors by pharmacy staff and the patient, handing over of comprehensible information material, and teach-back evaluation were the key components for effective counseling. In particular, periodic observations of individual administration skills seemed crucial to sustained teaching success. However, some patients probably require further error prevention strategies such as additional education materials or external support with eye-drop administration.

## Supporting information

S1 Fig Study design consisting of three follow-up visits one month, six months, and twelve months after the first encounter. The specific procedure comprised an assessment of administration skills, tailored counseling, and teach-back. At the first encounter, patients, who administered their drugs for the first time, were immediately counseled without baseline assessment of administration skills. Patients, who demonstrated correct administration at follow-up visits, received no further counseling at that visit.
(TIF)

S2 Fig Self-evaluation of administration skills prior to baseline skills assessment. Patients who regularly used eye-drops could rate their administration skills as “very good” (black), “good” (squared), “sometimes good, sometimes poor” (grey), “less good” (hatched), “not good” (striped), or “I don’t know” (white).
NP: non-participating patients, i.e., patients who correctly administered eye-drops and, thus, received no counseling or patients who received patient education but refused to participate in the follow-up visits.
(TIF)

S3 Fig Error profile of intervention patients who completed both follow-up visits (N = 15). Graphs are ordered according to number of errors and recurrence of already resolved errors at the 1-month or 6-month follow-up. Numbers indicated erroneous administration steps: 1 = hand washing, 2 = instilling a single drop, 3 = instillation into the conjunctival sac, 4 = eyelid closure for approximately one minute, 5 = nasolacrimal occlusion, 6 = dropper tip was not touched.
(TIF)

S4 Fig Error profile of control patients who completed both follow-up visits (N = 29). Graphs are ordered according to number of errors and recurrence of already resolved errors at the 1-month or 6-month follow-up. Numbers indicated erroneous administration steps: 1 = hand washing, 2 = instilling a single drop, 3 = instillation into the conjunctival sac, 4 = eyelid closure for approximately one minute, 5 = nasolacrimal occlusion, 6 = dropper tip was not touched.
(TIF)

S1 Table Duration of patient education at first encounter and follow-up visits. (PDF)

S2 Table Patients with correct eye drop administration before and after counseling. (PDF)

S1 File Trial protocol. (PDF)

S2 File Studienprotokoll. (PDF)

S3 File CONSORT 2010 checklist. (PDF)
