# Supplementary figures and images for: Improving eye-drop administration skills of patients – A multicenter parallel-group cluster-randomized controlled trial
Source: PLoS One. 2019 Feb 21;14(2):e0212007. doi: 10.1371/journal.pone.0212007 (PMC6383939; doi:10.1371/journal.pone.0212007)

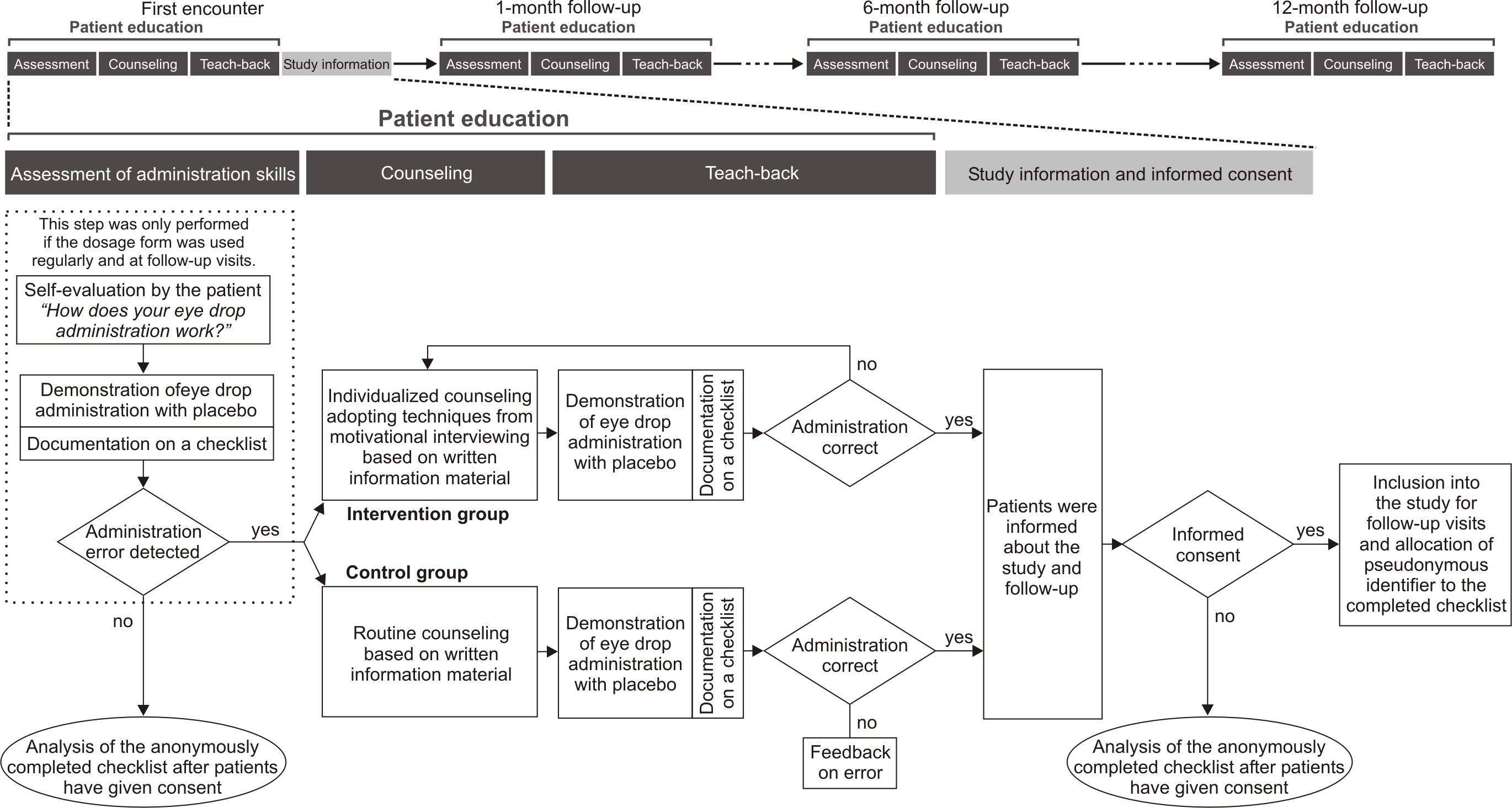

Supplement: S1 Fig — The specific procedure comprised an assessment of administration skills, tailored counseling, and teach-back. At the first encounter, patients, who administered their drugs for the first time, were immediately counseled without baseline assessment of administration skills. Patients, who demonstrated correct administration at follow-up visits, received no further counseling at that visit. (TIF) [file pone.0212007.s001.tif]

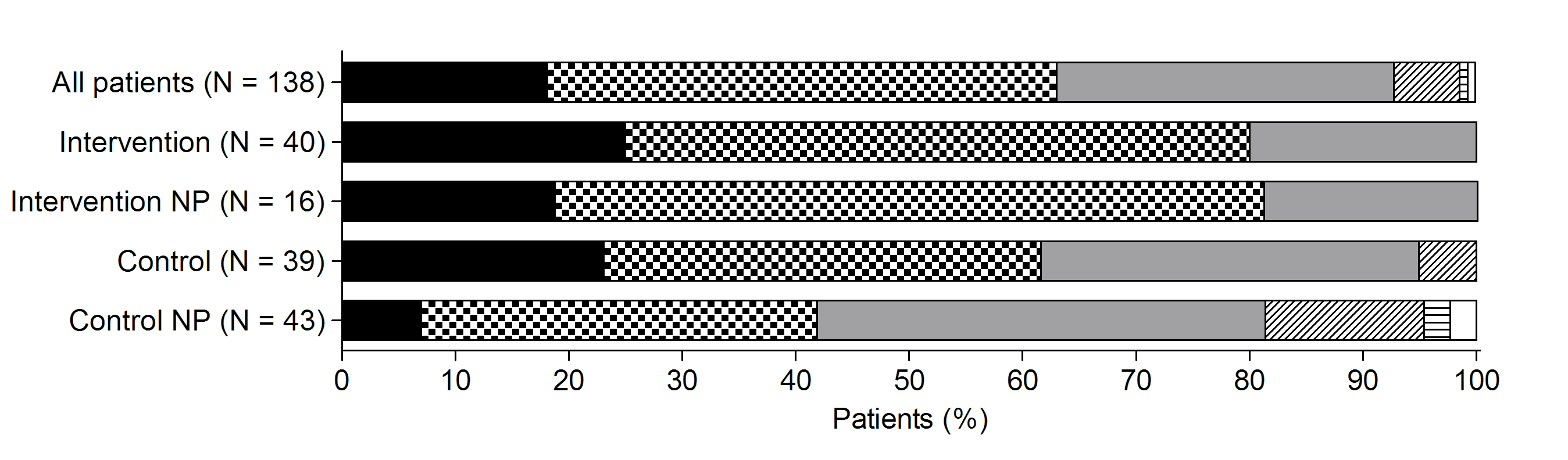

Supplement: S2 Fig — Patients who regularly used eye-drops could rate their administration skills as “very good” (black), “good” (squared), “sometimes good, sometimes poor” (grey), “less good” (hatched), “not good” (striped), or “I don’t know” (white). NP: non-participating patients, i.e., patients who correctly administered eye-drops and, thus, received no counseling or patients who received patient education but refused to participate in the follow-up visits. (TIF) [file pone.0212007.s002.tif]

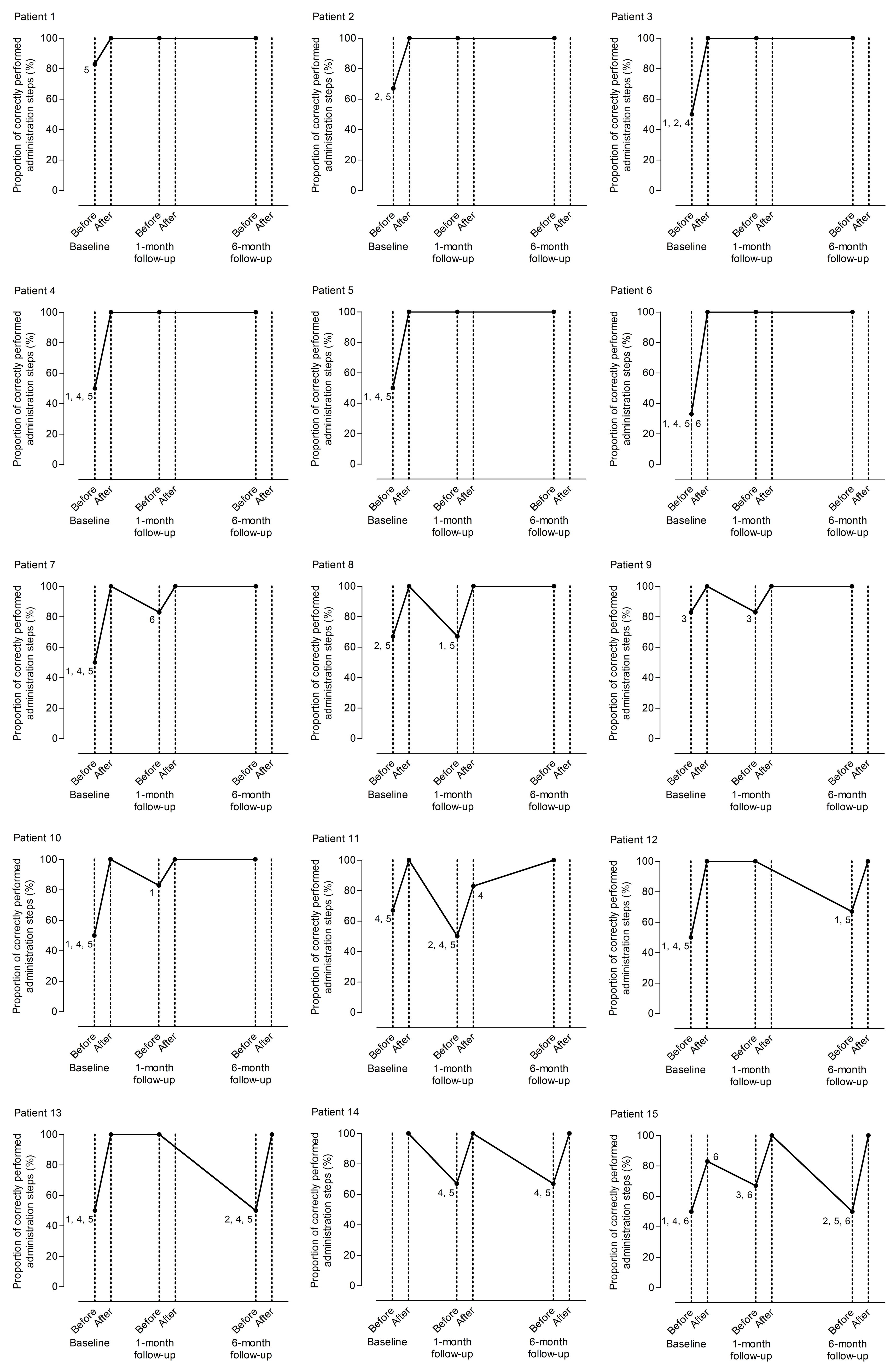

Supplement: S3 Fig — Graphs are ordered according to number of errors and recurrence of already resolved errors at the 1-month or 6-month follow-up. Numbers indicated erroneous administration steps: 1 = hand washing, 2 = instilling a single drop, 3 = instillation into the conjunctival sac, 4 = eyelid closure for approximately one minute, 5 = nasolacrimal occlusion, 6 = dropper tip was not touched. (TIF) [file pone.0212007.s003.tif]
