# Supplementary material for: Improving eye-drop administration skills of patients – A multicenter parallel-group cluster-randomized controlled trial
Source: PLoS One. 2019 Feb 21;14(2):e0212007. doi: 10.1371/journal.pone.0212007 (PMC6383939; doi:10.1371/journal.pone.0212007)
Supplement: S1 Table — (PDF) [file pone.0212007.s005.pdf]

S1 Table. Duration of patient education at first encounter and follow-up visits.

	**Intervention**	**Control**
First encounter (mean ± SD)		
First-time users	11 ± 5 min (N = 9) (range 5 to 20 min)	11 ± 7 min (N = 13) (range 3 to 25 min)
Regular users	13 ± 6 min (N = 49) (range 1 to 32 min)	10 ± 5 min (N = 81) (range 2 to 30 min)
1-month follow-up (mean ± SD)	9 ± 6 min (N = 30) (range 2 to 30 min)	10 ± 6 min (N = 39) (range 3 to 20 min)
6-month follow-up (mean ± SD)	10 ± 8 min (N = 15) (range 2 to 30 min)	8 ± 5 min (N = 29) (range 2 to 20 min)

SD = standard deviation
