# Supplementary material for: Improving eye-drop administration skills of patients – A multicenter parallel-group cluster-randomized controlled trial
Source: PLoS One. 2019 Feb 21;14(2):e0212007. doi: 10.1371/journal.pone.0212007 (PMC6383939; doi:10.1371/journal.pone.0212007)
Supplement: S2 Table — (PDF) [file pone.0212007.s006.pdf]

S2 Table. Patients with correct eye drop administration before and after counseling.

	**Baseline assessment (regular use)**			**Assessment after counseling (regular use)**			**Assessment after counseling (first use)**		
	**All (N = 138)**	Intervention (N = 56)	Control (N = 82)	**All N = 130)**	Intervention (N = 49)	Control (N = 81)	**All (N = 22)**	Intervention (N = 9)	Control (N = 13)
Number of patients who performed all steps correctly N (%)	**8 (6 %)**	7 (13 %)	1 (1 %)	**110 (85 %)**[a]	38 (78 %)[a]	72 (89 %)[a]	**12 (54 %)**	6 (67 %)	6 (46 %)
Number of patients who performed single steps correctly N (%)									
1. Hand washing	**50 (36 %)**	21 (38 %)	29 (35 %)	**126 (97 %)**[a]	46 (94 %)[a]	80 (99 %)[a]	**18 (82 %)**	8 (89 %)	10 (77 %)
2. Instilling a single drop	**108 (78 %)**	50 (89 %)	58 (71 %)	**127 (98 %)**[a]	48 (98 %)	79 (98 %)[a]	**20 (91 %)**	8 (89 %)	12 (92 %)
3. Instillation into the conjunctival sac	**110 (80 %)**	47 (84 %)	63 (77 %)	**128 (98 %)**[a]	47 (96 %)[a]	81 (100 %)	**21 (95 %)**	8 (89 %)	13 (100 %)
4. Eyelid closure for approximately one minute	**32 (23 %)**	18 (32 %)	14 (17 %)	**126 (97 %)**[a]	48 (98 %)[a]	78 (96 %)[a]	**18 (82 %)**	8 (89 %)	10 (77 %)
5. Nasolacrimal occlusion	**22 (16 %)**	16 (29 %)	6 (7 %)	**125 (96 %)**[a]	47 (96 %)[a]	78 (96 %)[a]	**15 (68 %)**	8 (89 %)	7 (54 %)
6. Dropper tip was not touched	**98 (71 %)**	46 (82 %)	52 (63 %)	**120 (92 %)**[a]	45 (92 %)[a]	75 (93 %)[a]	**22 (100 %)**	9 (100 %)	13 (100 %)

[a]significant compared to baseline ($p < 0.05$)
