# Supplementary material for: Improving eye-drop administration skills of patients – A multicenter parallel-group cluster-randomized controlled trial
Source: PLoS One. 2019 Feb 21;14(2):e0212007. doi: 10.1371/journal.pone.0212007 (PMC6383939; doi:10.1371/journal.pone.0212007)
Supplement: S1 File — (PDF) [file pone.0212007.s007.pdf]

Project plan

Evaluation of individualized education of patients and family caregivers on correct medication administration in community pharmacies

(Project number: K406)

Head of the project:
Dr. sc. hum. Hanna M. Seidling,
Head of the Cooperation Unit Clinical Pharmacy
Department of Clinical Pharmacology and Pharmacoepidemiology
Heidelberg University
Im Neuenheimer Feld 410
69120 Heidelberg
Phone: 06221 56 38736
Email: hanna.seidling@med.uni-heidelberg.de

Prof. Dr. med. Walter E. Haefeli
Head of the department of Clinical Pharmacology and Pharmacoepidemiology
Heidelberg University
Im Neuenheimer Feld 410
69120 Heidelberg
Phone: 06221 56 8740
Email: walter.emil.haefeli@med.uni-heidelberg.de

Coordination of the project:
Anette Lampert, Pharmacist
Department of Clinical Pharmacology and Pharmacoepidemiology
Cooperation Unit Clinical Pharmacy

Biometrician:
Dr. sc. hum. Thomas Bruckner
Institute of Medical Biometry and Informatics

Funding:
Anette Lampert received a personal scholarship from the „Dr. August and Dr. Anni Lesmüller foundation".

Date and version of the project plan:
Version 3, 05.12.2014

1. Summary

Correct prescription, dispensing and administration of drugs are crucial for safe pharmacotherapy. In hospitals, almost every fifth drug dose is administered erroneously. It is supposed that at home, where patients or family caregivers are responsible for correct drug administration, error rates are even higher. Patient education on correct medication administration increases adherence and safety of administration. When comparing different educational interventions, it became evident that they differ significantly in their effectiveness. Training that addresses the individual needs of the patient is more effective than interventions that provide general information. A previous focus group study with patients, family caregivers, and nurses (ethics vote S-206/2013), which aimed at determining drug administration problems, revealed that many medicine users were unaware of drug administration errors. To address the lack of error awareness, the Transtheoretical Model developed by James D. Prochaska and his colleagues provides a theoretical framework. This model involves five different levels that a person goes through when he intrinsically changes his behavior. Studies to improve compliance show that level-specific measures, according to the Transtheoretical Model, positively influence the outcome of an intervention. Therefore, this prospective randomized controlled study, aims to evaluate standardized patient education in community pharmacies. This study takes into account the possibly lacking error awareness of the patient or family caregiver and feasibility in community pharmacies. Pharmacy staff will be trained on the study procedure, use of study material and correct administration of different dosage forms. Subsequently, pharmacy staff includes patients during their daily routine into the study. In a before and after comparison of the intervention group with the control group and follow-up visits, the effectiveness and sustainability of patient education will be evaluated

2. Table of contents

1. Summary 2
2. Table of contents 3
3. Introduction 4
4. Aims and Issues of the project 6
5. Method to be tested 7
6. Study design 7
7. Planned sample size 7
8. Inclusion criteria 7
9. Exclusion criteria 8
10. Method of randomization 8
11. Study procedure 8
12. Concomitant therapy 11
13. Security laboratory 11
14. Stopping criteria 11
15. Statistical analyses 11
16. Ethical and legal aspects 12
17. Signatures 15
18. References 16

3. Introduction

The ideal pharmacotherapy does not only depend on choosing the right drug but also on an assured quality of prescription, dispensing and application. Notably incorrect medication administration therefore poses a current problem regarding safety of drug therapy [1-3]. At hospitals almost every fifth dose is administered incorrectly [1,2], either decreasing the efficacy of pharmacotherapy [4-7] or increasing the risk of an adverse drug event [3,4,8]. Out of hospital, it is rarely the ambulatory nursing service but more often the patient himself or family caregivers who are responsible for the medication administration. Thus, one can assume that in daily usage of drugs at home, the amount of incorrectly administered doses is even higher [9-11]. If the patient is overstrained due to medication administration this will frequently cause non-adherence [12,13]. Interventions (based on different training methods) for the correct administration of drugs could increase medication adherence and safety in patients and family caregivers as well as in health care professionals [14,15]. Comparing different training methods, it is shown that their effectiveness can differ noticeably [16]. Hereby, trainings considering individual patient's needs are more effective than methods educating more general information [16].

In a previous focus group study (ethics vote S-206/2013) for patients, family caregivers and nursing staff focusing on registration of medication administration problems in daily life and on determination of training requirements, it is shown that a lot of medicine users are not aware of possible errors [Lampert et al., unpublished]. In fact, patients often feel a lot better informed than they really are [17]. In a study about the correct application of eye drops, 93 % of the patients reported not to have problems with the application though this was indeed true for less than a third of the patients [18]. Just as 94 % of COPD patients overestimated their skills of administering their inhaler correctly [19]. The lack of error awareness makes it difficult to motivate such patients participating in training or a detailed consultation. The Transtheoretical Model developed by James D. Prochaska and his colleagues delivers the theoretical framework for considering the initially missing awareness for the problem on the way to change the behavior [20,21]. The Transtheoretical Model mainly includes five different levels a person will undergo if he or she changes his or her behavior [20]:

- Level 1: During the unintentional state (“Pre-contemplation”) persons do not follow the intention to change problematic behavior.
- Level 2: During the state of establishing intention (“Contemplation”) persons follow the intention to change problematic behavior eventually.
- Level 3: During the state of preparation (“Preparation”) persons plan precisely to change their problematic behavior soon and take first steps to the direction of altering their behavior.
- Level 4: During the state of action (“Action”) persons change their behavior.
- Level 5: During the state of maintenance (“Maintenance”) persons have given up their problematic behavior for a longer period of time.

With methods respecting the individual level of the patients or family caregivers, progress to change their behavior may be positively influenced [20]. Thus, studies for improving the compliance show that considering the individual levels influences the outcome of the intervention positively [21]. The model has not been applied yet for trainings to avoid medication errors. Yet it seems to be obvious that patients or family caregivers, who do not realize a medication error because they have not been aware of it, do not recognize the necessity for training. To reach these patients and family caregivers and to motivate them for training, the Transtheoretical Model could be the theoretical framework for implementing training interventions.

According to this prospective randomized controlled study, standardized patient education in community pharmacies should be evaluated. The training method should consider the patients’ or family caregivers’ lack of awareness if necessary and particularly should be integrated in the daily routine of pharmacies. For this reason, the pharmacy staff of the particular community pharmacy will carry out the training and document any additional work required. Before the study is started, the pharmacy staff will be informed about incorrect and correct administration of the dosage form which is known from literature (see inclusion criteria), the use of education materials and the study procedure. There are three follow-up visits planned, approximately one month, six months and twelve months after the first patient education. Patients or family caregivers will either be addressed in the community pharmacy (recruiting and follow-up visits) or they will be contacted by employees of the participating community pharmacies or by an employee of the department of Clinical Pharmacology and Pharmacoepidemiology and they will

be invited into the correspondent community pharmacy (follow-up visits). The pharmacy staff or employees of the department of Clinical Pharmacology and Pharmacoepidemiology will again assess the quality of medication administration of patients or family caregivers during their follow-up visits to evaluate the sustainability of the first training.

4. Aims and issues of the project

Primary outcome

- Rate of incorrect medication administration will be compared between control and intervention group at 6-month follow-up.

Secondary outcomes

- Rate of incorrect medication administration before and after patient education in the control and intervention group.
- Rate of incorrect medication administration during the course of follow-up visits.
- Influence of the consideration of the individual level according to the Transtheoretical Model [20] on the willingness to participate in a training, i.e. long-term (1, 6 and 12 months) correct drug use.
- Univariate and multivariate analyses to evaluate the influence of covariates such as dosage form, patient characteristics, the training person and the number of medicines on training success.
- Qualitative: feasibility of implementation in community pharmacies, satisfaction of patients and caregivers.

The following general questions are related to the project:

- Is it possible to motivate patients or family caregivers to attend training by raising awareness of a drug administration error?
- Is the consideration of the individual awareness of the error an advantage in the training compared to a "normal" counseling?
- Is the training success sustainable?
- Is the patient education intervention feasible in the community pharmacy setting?
- Are any differences in training success depending on the dosage form or person?

5. Method to be tested

General description

The influence of the individualization of patient education on the success of the training should be evaluated.

Effects/risks

Adverse events due to participation in the study are not expected. If relevant issues outside of drug use are identified in individual cases, assistance and advice on specific issues will be provided by pharmacy staff. If a misuse of the drug occurs during the trial, pharmacy staff will inform the patient or caregiver of the error and the correct application.

6. Study design

Prospective, cluster-randomized, controlled interventional study.

7. Planned sample size

See statistical analyses

8. Inclusion criteria

Patients and family caregivers:

- Age >18 years
- Physical and cognitive ability to attend training
- Application of at least one of the following dosage forms to oneself or others: Eye drops, transdermal patches, tablets to divide, liquid oral preparations and/or capsules
- Written informed consent to study participation after oral and written clarification

Pharmacy staff:

- Written informed consent to study participate in the study after clarification in a written or oral form

Community pharmacies:

- Spatial requirements to enable confidential consultation

9. Exclusion criteria

Minors or persons who are not able to give their consent or who are physically or cognitively unable to attend training or understand German education are excluded. Also excluded are study participants (patients, caring family members and pharmacy staff) who do not give or withdraw their written informed consent.

10. Method of randomization

The participating community pharmacies are continuously assigned to a randomization plan drawn up in advance. The randomization plan is created online at www.randomization.com

11. Study procedure

The study will be carried out in community pharmacies with which other projects have already established cooperation. Prior to the start of the study, pharmacy staff will be informed of erroneous and correct administration of the different dosage forms (see inclusion criteria) known from the literature, the use of study materials, and the study procedure. After written informed consent of the head of the pharmacy and pharmacy staff, the pharmacies will be assigned to control or intervention group (cluster randomization). There will be exclusive control pharmacies and intervention pharmacies.

Pharmacy staff addresses potential study participants according to the inclusion criteria during daily routine in the community pharmacy. It is possible within the scope of this study to train the use of different dosage forms for the same patient or family caregiver. In order to identify a potential administration error, the pharmacy staff asks the patient or family caregiver to use the medicine (duration approx. 2-5 minutes). Such a demonstration is quite common in the context of the usual pharmaceutical counseling. The demonstration can be performed with the patient's drug itself or with a placebo. The pharmacy staff assesses a possibly incorrect administration by using a checklist defined in advance for a specific dosage form. Checklists and placebo materials are provided by the Department of Clinical Pharmacology and Pharmacoepidemiology. In the control group, pharmacy staff provides a standard consultation (duration approx. 3 minutes) followed by the provision of standardized information material, e.g. written step-by-step

instructions. In the intervention group, the staging algorithm (Fig. 1) is used to determine the individual level of the patient or caregiver according to the Transtheoretical Model. The stage "Maintenance", i.e. the permanent correct use of the drug, cannot be detected with this algorithm. The purpose of this model is to identify in particular those medicines users who are not aware of administration errors despite incorrect use of the drug and who may not be motivated to seek training or detailed counseling.

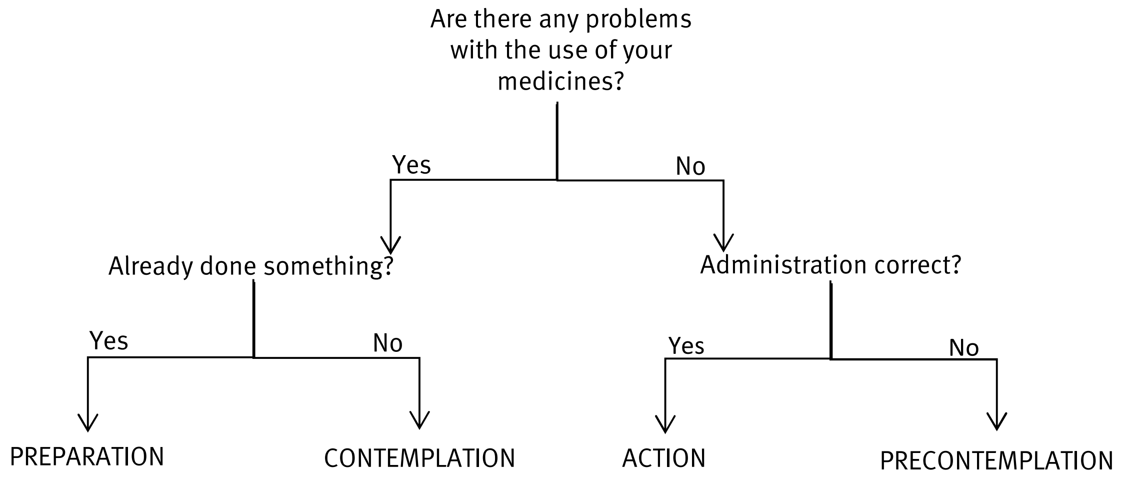

Figure 2. Staging Algorithm

Patients or caregivers who are in a “pre-action stage” (i.e., precontemplation, contemplation, and preparation) will be informed and trained by the pharmacy staff about the use of the drug according to their individual level (Table 1) (approximately 5-10 minutes).

Table 1. Possible level-specific interventions.

**Stage of change**	**Study-specific definition**	**Possible intervention**	**Example**
Pre-contemplation	Lack of error awareness	e.g. create awareness of the problem, point out risks of incorrect application	Presentation of a risk score, information about possible consequences of incorrect application and advantages of correct application
Contemplation	Aware of an error	e.g. point out the risks of incorrect application, show possibilities	Proposal for a training

Preparation	Aware of an error, search for solutions	e.g. standardized education materials for specific dosage forms	Education based on standardized information materials
Action	Drug is administered correctly	e.g. regular monitoring	Follow-up visits with re-presentation of the application, assessment by the pharmacy staff and retraining
Maintenance	Drug is permanently administered correctly	e.g. regular monitoring	Follow-up visits with re-presentation of the application and assessment by the pharmacy staff

Immediately after the training (intervention group) or after issuing the standardized instructions (control group), the drug application is checked again using the checklist. If errors continue to occur, there will be a corresponding retraining in the intervention group. In the control group, the patient or family caregiver is made aware of the errors verbally.

After the pharmaceutical consultation that can represent a standard situation in community pharmacies, the patients or caregivers are invited to participate in the follow-up visits Study information is provided by the pharmacy staff or staff of the department of Clinical Pharmacology and Pharmacoepidemiology. If the patient or caregiver wishes to participate in the study and attests this by his written consent, his so far anonymized data will be pseudonymized. After inclusion in the study, patients and caregivers are asked to answer a sociodemographic questionnaire (duration approx. 5 minutes). In addition, e.g. finger strength and cognitive abilities may be considered as factors influencing the training success. Further data collection during follow-up visits is pseudonymized. Within the study, three follow-up visits will be planned at 1 month, 6 months and 12 months. Patients or caregivers are either referred to the follow-up at the pharmacy at the appropriate time or contacted by pharmacy staff or staff of the department of Clinical Pharmacology and Pharmacoepidemiology. Patients or caregivers can choose the preferred way of contacting, e.g. call, email or letter, prior to inclusion into the study. At follow-up visits the patient or family caregiver will be asked to demonstrate again drug administration, which is evaluated by the pharmacy staff or staff of the department of Clinical Pharmacology and Pharmacoepidemiology using the checklist. In the case of incorrect application, re-training takes place again. During the follow-up visits, patients and caregivers might be contacted by pharmacy staff or staff of the department of Clinical Pharmacology and Pharmacoepidemiology, e.g. by

telephone or in writing by e-mail or post to assess the satisfaction of the participants with regard to the study. The study procedure will be evaluated and, if necessary, adjusted in a pilot phase with regard to feasibility by study staff and pharmacy staff.

12. Concomitant therapy

Not applicable

13. Security laboratory

Not applicable

14. Stopping criteria

Individual

The approval of the study participants (patients, family caregivers and pharmacy staff) can be withdrawn at any time without indicating reasons and without any disadvantages.

Total study

The study will be discontinued if, contrary to the expectations, a potential risk should exist for the study participants.

15. Statistical method

Statistical design

The error rate of the intervention group will be compared to the error rate of the control group by the chi-square test. The error rate of the medication administration before patient education will be compared to the error rate after the training by the McNemar test. A p-value ≤ 0.05 is considered as statistically significant. Feasibility of the education method into the daily routine of the community pharmacy and the contentment of the patients and family caregivers will be analyzed qualitatively. The influence of co-variables like dosage form, patient characteristics, educating person and number of drugs on the education success will be determined by logistic regression.

Calculation of sample size based on the primary outcome

Due to the results of a randomized study which has compared a complex education intervention for the correct application of inhalers with a standard consultation, we presume a difference for the success of the intervention in the control- and intervention groups of 20%, with success rates of p1=32% and p2=52% [22]. To determine a significant difference between the control- and intervention group by the chi-square test (unrelated sample, $\alpha = 0.05$; $\beta = 0.8$), it is required, in due consideration of a drop-out rate of 15% after 6 months, a cluster size of 5 community pharmacies per group and a cluster effect (Intracluster Correlation Coefficient, iCC) of 0.02, n=122 medication administrations of a dosage form e.g. eye drops or transdermal patches per group. With a cluster size of 10 community pharmacies per group the sample size of the dosage form increases to n=133 per group. The calculation of the sample size is carried out on the level of dosage forms because then patients who apply for various dosage forms can be educated according to their individual drugs. The follow-up visit after 12 months will be described qualitatively.

16. Ethical and legal aspects

The study is conducted according to the current version of the Declaration of Helsinki of the World Medical Association and the ethical principles for research on humans.

The study protocol will be submitted to the responsible ethics committee of the medical faculty Heidelberg for evaluation before the beginning of the study. It will not be started with the inclusion of study participants unless the written affirmative vote of this ethics committee is available.

Until the inclusion of the patients or their family members for the follow-up, the study serves to ensure quality assurance of the consultation quality of the pharmacy staff in the participating community pharmacies. The demonstration of the application by the medication user and the following consultation by the pharmacist can already take place during routine counseling. The pharmacy staff will document the work load within this quality-assuring project, for example additional time effort or interruption of work routine for evaluating the feasibility for daily use of the training. If patients or their family caregivers do not agree to participate in the study, the data on medication administration that was collected during the consultation might be used for

evaluation of the consulting quality. Pseudonymization does not happen because there is no follow-up for these patients or their family caregivers.

The participation of all participants in this study is voluntary.

The pharmacy staff of the participating community pharmacies will be informed in written and oral form before the beginning of the study about the nature and scope of the planned intervention, in particular about the possible benefits and risks or burdens, the voluntariness of the participation and about the required documentation of their approval by signing the declaration of consent. To cluster the educated medication users not only according to the community pharmacy but also according to the educating person, the pharmacy staff will get a pseudonym which indicates their profession (e.g. pharmacist or pharmacy technician) and their professional experience (less than a year, 1-5 years, more than 5 years).

The patients and family caregivers will be informed in written and oral form after the training about the nature and scope of the planned follow-up visits, about the possible benefits and risks or burdens, the voluntariness of their participation and about the documentation of their approval by signing the declaration of consent.

The information document informs the study participants (patients, family caregivers and pharmacy staff) about data collection which is carried out in a pseudonymized form. Furthermore, patients and family caregivers will be informed that the data collected up to the time of inclusion will be pseudonymized when they are included in the study to enable an assignment of the follow-up visits. The pharmacy staff can only transfer the corresponding patient identification number to the data collected up to this moment if the patient agrees immediately after the conducted consultation and data collection to participate in the follow-up visits. If the patient is not able to do that, he will not be included in the study. At the time of scientific publication, data are pseudonymized for the staff of the department of Clinical Pharmacology and Pharmacoepidemiology, and the data is actually anonymized for third persons at any time. The approval of the participants of the study can be withdrawn at any time without declaring any reasons and without any disadvantages for further medical treatment. If the approval is withdrawn, it will be decided together with the study participant whether he agrees to the evaluation of the collected data up to this moment; otherwise his data will be deleted. If individual members of the pharmacy staff withdraw their consent, the study can be continued in the pharmacy with the pharmacy staff still participating.

The names of the study participants and every confidential information is bound to professional discretion according to the penal code (StGB), the professional codes of discretion and the regulations of the German Data Protection Act (BDSG).

For the evaluation, data (e. g. names and other personal information) will be pseudonymized. Data of participants will only be passed on in a pseudonymized form as the case may be. Third persons will not gain any insight into study documents which contain non-anonymized personal data. The results will be published in a consolidated way without indicating any names or other personal data of the participants. Data and documentation papers will be archived on the central secured server of Heidelberg University Hospital.

17. Signatures

Heidelberg, the 05.12.2014

Dr. sc. hum. Hanna M. Seidling

Heidelberg, the 05.12.2014

Prof. Dr. med. Walter E. Haefeli

Heidelberg, the 05.12.2014

Anette Lampert, pharmacist

18. References

1. Keers RN, Williams SD, Cooke J, Ashcroft DM. Prevalence and nature of medication administration errors in health care settings: a systematic review of direct observational evidence. Ann Pharmacother 2013;47:237-56.
2. Berdot S, Gillaizeau F, Caruba T, Prognon P, Durieux P, Sabatier B. Drug administration errors in hospital inpatients: a systematic review. PLoS One 2013;8:e68856.
3. Leape LL, Bates DW, Cullen DJ, Cooper J, Demonaco HJ, Gallivan T, Hallisey R, Ives J, Laird N, Laffel G. Systems analysis of adverse drug events. ADE Prevention Study Group. JAMA 1995;274:35-43.
4. Lampert A, Seiberth J, Haefeli WE, Seidling HM. A systematic review of medication administration errors with transdermal patches. Expert Opin Drug Saf 2014;13:1101-14.
5. Hansen B, Matytsina I. Insulin administration: selecting the appropriate needle and individualizing the injection technique. Expert Opin Drug Deliv 2011;8:1395-406.
6. Chowdhury TA, Escudier V. Poor glycaemic control caused by insulin induced lipohypertrophy. BMJ 2003;327:383-4.
7. Sleath B, Blalock S, Covert D, Stone JL, Skinner AC, Muir K, Robin AL. The relationship between glaucoma medication adherence, eye drop technique, and visual field defect severity. Ophthalmology 2011;118:2398-402.
8. Kale A, Keohane CA, Maviglia S, Gandhi TK, Poon EG. Adverse drug events caused by serious medication administration errors. BMJ Qual Saf 2012;21:933-8.
9. Walsh KE, Roblin DW, Weingart SN, Houlahan KE, Degar B, Billett A, Keuker C, Biggins C, Li J, Wasilewski K, Mazor KM. Medication errors in the home: a multisite study of children with cancer. Pediatrics 2013;131:e1405-14.
10. Mager DR. x
11. Kripalani S, Roumie CL, Dalal AK, Cawthon C, Businger A, Eden SK, Shintani A, Sponsler KC, Harris LJ, Theobald C, Huang RL, Scheurer D, Hunt S, Jacobson TA, Rask KJ, Vaccarino V, Gandhi TK, Bates DW, Williams MV, Schnipper JL, PILL-CVD (Pharmacist Intervention for Low Literacy in Cardiovascular Disease) Study Group. Effect of a pharmacist intervention on clinically important medication errors after hospital discharge: a randomized trial. Ann Intern Med 2012;157:1-10.
12. Winfield AJ, Jessiman D, Williams A, Esakowitz L. A study of the causes of non-compliance by patients prescribed eyedrops. Br J Ophthalmol. 1990;74:477-80.
13. Ryan R, Santesso N, Hill S, Lowe D, Kaufman C, Grimshaw J. Consumer-oriented interventions for evidence-based prescribing and medicines use: an overview of systematic reviews. 2011:CD007768.
14. Bertsche T, Bertsche A, Krieg EM, Kunz N, Bergmann K, Hanke G, Hoppe-Tichy T, Ebinger F, Haefeli WE. Prospective pilot intervention study to prevent medication errors in drugs administered to children by mouth or gastric tube: a programme for nurses, physicians and parents. Qual Saf Health Care 2010;19:e26.
15. Sullivan MM, O'Brien CR, Gitelman SE, Shapiro SE, Rushakoff RJ. Impact of an interactive online nursing educational module on insulin errors in hospitalized pediatric patients. Diabetes care. 2010;33:1744-6.
16. Friedman AJ, Cosby R, Boyko S, Hatton-Bauer J, Turnbull G. Effective teaching strategies and methods of delivery for patient education: a systematic review and practice guideline recommendations. J Cancer Educ 2011;26:12-21.
17. Brounéus F, Macleod G, Maclennan K, Parkin L, Paul C. Drug safety awareness in New Zealand: public knowledge and preferred sources for information. J Prim Health Care 2012;4:288-93.
18. Stone JL, Robin AL, Novack GD, Covert DW, Cagle GD. An objective evaluation of eyedrop instillation in patients with glaucoma. Arch Ophthalmol 2009;127:732-6.
19. Souza ML, Meneghini AC, Ferraz E, Vianna EO, Borges MC. Knowledge of and technique for using inhalation devices among asthma patients and COPD patients. J Bras Pneumol 2009;35:824-31.
20. Prochaska JO, Velicier WF. The transtheoretical model of health behavior change. Am J Health Promot 1997;12:38-48.
21. Ficke DL, Farris KB. Use of the transtheoretical model in the medication use process. Ann Pharmacother 2005;39:1325-30.
22. Press VG, Arora VM, Shah LM, Lewis SL, Charbeneau J, Naureckas ET, Krishnan JA. Teaching the use of respiratory inhalers to hospitalized patients with asthma or COPD: a randomized trial. J Gen Intern Med 2012;27:1317-25.
