# Supplementary material for: Improving eye-drop administration skills of patients – A multicenter parallel-group cluster-randomized controlled trial
Source: PLoS One. 2019 Feb 21;14(2):e0212007. doi: 10.1371/journal.pone.0212007 (PMC6383939; doi:10.1371/journal.pone.0212007)
Supplement: S2 File — (PDF) [file pone.0212007.s008.pdf]

Projektplan

Evaluation einer individualisierten Schulungsmaßnahme zur richtigen Arzneimittelanwendung durch Patienten und pflegende Angehörige in öffentlichen Apotheken

(Projektnummer: K406)

Leiter des Projekts:
Dr. sc. hum. Hanna M. Seidling,
Leiterin Kooperationseinheit Klinische Pharmazie
Abt. Klinische Pharmakologie und Pharmakoepidemiologie
Medizinische Klinik der Universität Heidelberg
Im Neuenheimer Feld 410
69120 Heidelberg
Tel. Nr.: 06221 56 38736
Email: hanna.seidling@med.uni-heidelberg.de

Prof. Dr. med. Walter E. Haefeli
Ärztlicher Direktor
Abt. Klinische Pharmakologie und Pharmakoepidemiologie
Medizinische Klinik der Universität Heidelberg
Im Neuenheimer Feld 410
69120 Heidelberg
Tel. Nr.: 06221 56 8740
Email: walter.emil.haefeli@med.uni-heidelberg.de

Projektkoordination:
Anette Lampert, Apothekerin
Abt. Klinische Pharmakologie und Pharmakoepidemiologie
Kooperationseinheit Klinische Pharmazie

Biometriker:
Dr. sc. hum. Thomas Bruckner
Institut für Medizinische Biometrie und Informatik
Abteilung Medizinische Biometrie

Sponsor:
Die Studie findet im Rahmen eines Promotionsstipendiums für Frau Anette Lampert (persönliche Förderung, keine Projektförderung) der „Dr. August und Dr. Anni Lesmüller-Stiftung“ statt.

Datum und Version des Projektplans:
Version 3, 05.1210.2014

1. Zusammenfassung

Für eine sichere Pharmakotherapie ist neben der Wahl des geeigneten Arzneimittels eine gesicherte Verordnungs-, Abgabe- und Anwendungsqualität essentiell. Beinahe jede fünfte Dosis wird im Krankenhaus fehlerhaft verabreicht. Es ist zu vermuten, dass im häuslichen Umfeld, wo zumeist der Patient selbst oder ein pflegender Angehöriger und nur selten ein ambulanter Pflegedienst für die Arzneimittelapplikation verantwortlich ist, die Anzahl der fehlerhaft verabreichten Dosen höher ist. Schulungen der korrekten Arzneimittelanwendung konnten sowohl bei Patienten als auch pflegenden Angehörigen die Adhärenz und Sicherheit der Arzneimittelanwendung erhöhen. Beim Vergleich unterschiedlicher Schulungsmaßnahmen zeigt sich jedoch, dass diese sich in ihrer Effektivität deutlich unterscheiden können. Dabei sind Schulungen, die individuelle Bedürfnisse des Patienten berücksichtigen, effektiver als Maßnahmen, die allgemeine Informationen schulen. In einer vorausgegangenen Fokusgruppen-Befragung (Ethik-Votum S-206/2013) bei Patienten, pflegenden Angehörigen und Pflegekräften zur Erfassung von Arzneimittelanwendungsproblemen im Alltag und Ermittlung des Schulungsbedarfs, zeigte sich, dass sich viele der Arzneimittelanwender über mögliche Probleme nicht bewusst sind. Um das ggf. fehlende Problembewusstsein zu berücksichtigen bietet das von James D. Prochaska und seinen Kollegen entwickelte Transtheoretische Modell einen theoretischen Rahmen. Im Kern umfasst dieses Modell fünf unterschiedliche Stufen, die ein Mensch durchläuft, wenn er intrinsisch sein Verhalten ändert. Studien zur Verbesserung der Compliance zeigen, dass stufenspezifische Maßnahmen, gemäß dem Transtheoretischen Modell, den Ausgang einer Intervention positiv beeinflussen. Im Rahmen dieser prospektiven randomisierten kontrollierten Studie soll eine standardisierte Schulungsmaßnahme in öffentlichen Apotheken evaluiert werden, die das ggf. fehlende Problembewusstsein des Patienten oder pflegenden Angehörigen berücksichtigt und vor allem in den Apotheken-Alltag integrierbar ist. Nachdem das pharmazeutische Personal der teilnehmenden Apotheken durch Mitarbeiter der Abteilung Klinische Pharmakologie und Pharmakoepidemiologie über den Studienverlauf, Verwendung der Schulungsunterlagen und die korrekte Anwendung ausgewählter Darreichungsformen geschult wurde, wird die Studie durch das pharmazeutische Personal in den öffentlichen Apotheken durchgeführt. In einem Vorher-Nachher-Vergleich der Interventions-Gruppe mit der Kontroll-Gruppe sowie mithilfe von Follow-up-Besuchen soll die Effektivität und Nachhaltigkeit der Schulungsmaßnahme evaluiert werden.

2. Inhaltsverzeichnis

1. Zusammenfassung 2
2. Inhaltsverzeichnis 3
3. Einleitung 4
4. Ziele und Fragestellungen des Projekts 6
5. Zu prüfendes Verfahren 7
6. Studiendesign 7
7. Geplante Fallzahl 7
8. Einschlusskriterien 8
9. Ausschlusskriterien 8
10. Randomisierungsverfahren 8
11. Studienablauf 9
12. Begleittherapie 12
13. Sicherheitslabor 12
14. Abbruchkriterien 13
15. Statistisches Verfahren 13
16. Ethische und rechtliche Aspekte 14
17. Unterschriften 17
18. Referenzen 18

3. Einleitung

Die ideale Pharmakotherapie ist nicht nur von der Wahl des richtigen Arzneimittels, sondern auch von einer gesicherten Verordnungs-, Abgabe- und Anwendungsqualität abhängig. Insbesondere die fehlerhafte Arzneimittelanwendung stellt dabei ein aktuelles Problem in der Arzneimitteltherapiesicherheit dar [1-3]. Im Krankenhaus wird nahezu jede fünfte Dosis fehlerhaft verabreicht [1,2], was zum einen die Effektivität der Pharmakotherapie vermindern [4-7] und zum anderen das Risiko für unerwünschte Arzneimittelwirkungen erhöhen kann [3,4,8]. Außerhalb des Krankenhauses sind nur selten ein ambulanter Pflegedienst, häufiger jedoch der Patient selbst oder dessen Angehörige für die Arzneimittelanwendung verantwortlich, weshalb die Vermutung nahe liegt, dass im alltäglichen Gebrauch von Arzneimitteln außerhalb des Krankenhauses die Anzahl fehlerhaft applizierter Dosen sogar noch höher ist [9-11]. Überfordert die Arzneimittelanwendung den Patienten, hat dies häufig Non-Adhärenz zur Folge [12,13]. Interventionen (basierend auf unterschiedlichen Schulungsmethoden) für die korrekte Anwendung von Arzneimitteln konnten sowohl bei Patienten und pflegenden Angehörigen als auch bei medizinischem Personal die Adhärenz und Sicherheit der Arzneimittelanwendung erhöhen [14,15]. Beim Vergleich unterschiedlicher Schulungsmaßnahmen zeigt sich, dass diese sich in ihrer Effektivität deutlich unterscheiden können [16]. Dabei sind Schulungen, die individuelle Bedürfnisse des Patienten berücksichtigen, effektiver als Maßnahmen, die allgemeine Informationen schulen [16].

In einer vorausgegangenen Fokusgruppen-Befragung (Ethik-Votum S-206/2013) bei Patienten, pflegenden Angehörigen und Pflegekräften zur Erfassung von Arzneimittelanwendungsproblemen im Alltag und Ermittlung des Schulungsbedarfs, zeigte sich, dass sich viele der Arzneimittelanwender über mögliche Probleme nicht bewusst sind [Lampert et al., unveröffentlichte Daten]. Tatsächlich fühlen sich Patienten häufig besser informiert, als sie es in Wirklichkeit sind [17]. In einer Studie zur richtigen Anwendung von Augentropfen berichteten 93% der Patienten keine Probleme bei der Anwendung zu haben, allerdings traf dies tatsächlich auf weniger als ein Drittel der Patienten zu [18]. Ebenso überschätzten 94% der COPD-Patienten ihre Fähigkeit ihren Inhalator korrekt anzuwenden [19]. Durch das fehlende Problembewusstsein ist es schwierig, solche Patienten für die Teilnahme an einer Schulung oder ausführlichen Beratung zu motivieren. Das von James D. Prochaska und seinen Kollegen entwickelte Transtheoretische

Modell bietet den theoretischen Rahmen, um das anfangs fehlende Problembewusstsein auf dem Weg der Verhaltensänderung zu berücksichtigen [20,21]. Das Transtheoretische Modell umfasst im Kern fünf unterschiedliche Stufen, die ein Mensch durchläuft, wenn er intrinsisch sein Verhalten ändert [20]:

- Stufe 1: Im Absichtslosigkeitsstadium („Precontemplation") haben Personen keine Absicht, ein problematisches Verhalten zu verändern.
- Stufe 2: Im Absichtsbildungsstadium („Contemplation") haben Personen die Absicht, irgendwann das problematische Verhalten zu verändern.
- Stufe 3: Im Vorbereitungsstadium („Preparation") planen Personen konkret, demnächst ihr problematisches Verhalten zu ändern und unternehmen erste Schritte in Richtung einer Verhaltensänderung.
- Stufe 4: Im Handlungsstadium („Action") vollziehen Personen eine Verhaltensänderung.
- Stufe 5: Im Aufrechterhaltungsstadium („Maintenance") haben Personen seit einem längeren Zeitraum das problematische Verhalten aufgegeben.

Durch Maßnahmen, die die individuelle Stufe des Patienten oder pflegenden Angehörigen berücksichtigen, kann der Fortschritt in Richtung Verhaltensänderung positiv beeinflusst werden [20]. So zeigen Studien zur Verbesserung der Compliance, dass die Berücksichtigung der individuellen Stufen den Ausgang der Intervention positiv beeinflusst [21]. Das Model wurde bisher noch nicht für Schulungen zur Vermeidung von Arzneimittelanwendungsfehlern angewendet. Es liegt jedoch nahe, dass Patienten oder pflegende Angehörige, die einen Arzneimittelanwendungsfehler nicht als solchen erkennen, weil sie sich nicht darüber bewusst sind, dass ein Problem auftreten kann, auch keinen Schulungsbedarf erkennen. Um diese Patienten und pflegende Angehörige zu erreichen und für eine Schulung zu motivieren könnte das Transtheoretische Model als theoretischer Rahmen bei der Durchführung von Schulungsinterventionen dienen.

Im Rahmen dieser prospektiven randomisierten kontrollierten Studie soll eine standardisierte Schulungsmaßnahme in öffentlichen Apotheken evaluiert werden. Die Schulungsmaßnahme soll das ggf. fehlende Problembewusstsein des Patienten oder pflegenden Angehörigen berücksichtigen und vor allem in den Apotheken-Alltag integrierbar sein. Aus diesem Grund wird das pharmazeutische Personal der jeweiligen öffentlichen Apotheke die Schulung durchführen

und den ggf. zusätzlichen Arbeitsaufwand dokumentieren. Vor Beginn der Studie wird das pharmazeutische Personal über die aus der Literatur bekannte fehlerhafte und korrekte Anwendungspraxis der ausgewählten Darreichungsformen (siehe Einschlusskriterien), Verwendung der Schulungsunterlagen und den Studienablauf durch Mitarbeiter der Abteilung Klinische Pharmakologie und Pharmakoepidemiologie informiert. Es sind drei Follow-up-Besuche ca. 1 Monat, 6 Monate und 12 Monate nach der ersten Schulung geplant. Die Patienten oder pflegenden Angehörige werden entweder in der Apotheke angesprochen (Rekrutierung und Follow-up-Besuche) oder durch Mitarbeiter der teilnehmenden Apotheken oder einen Mitarbeiter der Abteilung Klinische Pharmakologie und Pharmakoepidemiologie kontaktiert und in die entsprechende Apotheke eingeladen (Follow-up-Besuche). Das pharmazeutische Personal oder Mitarbeiter der Abteilung Klinische Pharmakologie und Pharmakoepidemiologie beurteilen bei den Follow-up-Besuchen erneut die Qualität der Arzneimittelanwendung durch die Patienten oder pflegenden Angehörigen, um die Nachhaltigkeit der erfolgten Schulung zu evaluieren.

4. Ziele und Fragestellungen des Projekts

Hauptzielkriterien

- Rate fehlerhafter Arzneimittelanwendungen im Vergleich von Kontroll- und Interventionsgruppe zum Follow-Up Zeitpunkt 2.

Nebenzielkriterien

- Rate fehlerhafter Arzneimittelanwendungen vor und nach der Schulungsmaßnahme in Kontroll- und Interventionsgruppe
- Rate fehlerhafter Arzneimittelanwendungen im zeitlichen Verlauf der Follow-Up Erhebungen
- Einfluss der Berücksichtigung individueller Stufen gemäß des Transtheoretischen Modells [20] auf die Bereitschaft an einer Schulung prinzipiell bzw. erfolgreich teilzunehmen, d.h. langfristige (1, 6 und 12 Monate) korrekte Arzneimittelanwendung.
- Univariate und multivariate Analyse zur Evaluation des Einflusses von Kovariablen wie z.B. Darreichungsform, Patientencharakteristika, schulender Person und Anzahl der Arzneimittel auf den Schulungserfolg.

- Qualitativ: Integrierbarkeit in den Apotheken-Alltag, Zufriedenheit der Patienten und pflegenden Angehörigen.

Folgende übergeordnete Fragestellungen sind mit dem Projekt verbunden:

- Ist es möglich durch die Sensibilisierung für ein Arzneimittelanwendungsproblem Patienten oder pflegende Angehörige für eine Schulung zu motivieren?
- Ist die Berücksichtigung des individuellen Problembewusstseins ein Vorteil bei der Schulung im Vergleich zu einer „normalen“ Beratung?
- Bleibt der Schulungserfolg nachhaltig bestehen?
- Ist diese Schulungsmaßnahme in den Apotheken-Alltag integrierbar?
- Sind eventuelle Unterschiede im Schulungserfolg Darreichungsform-spezifisch und/oder Personen-spezifisch?

5. Zu prüfendes Verfahren

Allgemeine Beschreibung

Der Einfluss der Individualisierung einer Schulungsmaßnahme auf den Schulungserfolg soll evaluiert werden.

Wirkungen/Risiken

Unerwünschte Ereignisse durch die Teilnahme an der Studie sind nicht zu erwarten. Falls im Einzelfall relevante Probleme außerhalb der Arzneimittelanwendung identifiziert werden, wird Hilfestellung und Beratung zu spezifischen Fragestellungen durch Studienmitarbeiter angeboten. Fällt während der Studie eine fehlerhafte Arzneimittelanwendung auf, wird im Rahmen der pharmazeutischen Tätigkeit eingegriffen und der Patient oder pflegende Angehörige über den Fehler und die richtige Anwendung informiert.

6. Studiendesign

Prospektive, cluster-randomisierte, kontrollierte Interventionsstudie.

7. Geplante Fallzahl

siehe statistisches Verfahren

8. Einschlusskriterien

Patienten und pflegende Angehörige:

- Alter ≥18 Jahre
- Physische und kognitive Eignung zur Teilnahme an einer Schulung
- Anwendung von mindestens einer der folgenden Darreichungsformen bei sich selbst oder anderen: Augentropfen, Transdermale Therapeutische Systeme (TTS), Tabletten zum Teilen, flüssige perorale Zubereitungen und/oder Kapseln
- Schriftliche Einwilligung zur Studienteilnahme nach mündlicher und schriftlicher Aufklärung

Pharmazeutisches Personal:

- Schriftliche Einwilligung zur Studienteilnahme nach mündlicher und schriftlicher Aufklärung

Apotheken:

- Räumliche Voraussetzungen, um eine vertrauliche Beratung zu ermöglichen

9. Ausschlusskriterien

Ausgeschlossen werden Minderjährige oder Personen, die nicht einwilligungsfähig bzw. physisch oder kognitiv nicht in der Lage sind, an einer Schulung teilzunehmen oder eine deutsche Aufklärung zu verstehen. Ausgeschlossen werden darüber hinaus Studienteilnehmer (Patienten, pflegende Angehörige und pharmazeutisches Personal), die keine Einwilligungserklärung unterschreiben oder ihre Einwilligungserklärung zurückziehen.

10. Randomisierungsverfahren

Die Studien-Apotheken werden kontinuierlich einem vorab erstellten Randomisierungsplan zugeordnet. Der Randomisierungsplan wird online über www.randomization.com erstellt.

11. Studienablauf

Die Studie wird in öffentlichen Apotheken durchgeführt, mit denen bereits durch andere Projekte eine Zusammenarbeit etabliert ist. Vor Beginn der Studie wird das pharmazeutische Personal über die aus der Literatur bekannte fehlerhafte und korrekte Anwendungspraxis der ausgewählten Darreichungsformen (siehe Einschlusskriterien), Verwendung der Schulungsunterlagen und den Studienablauf durch Mitarbeiter der Abteilung Klinische Pharmakologie und Pharmakoepidemiologie informiert. Nach schriftlichem Einverständnis der entsprechenden Apotheken-Mitarbeiter werden die Apotheken einem vorab erstellen Randomisierungsplan folgend der Kontroll- oder Interventions-Gruppe zugeordnet (Clusterrandomisierung, Abb. 1). Damit wird es ausschließliche Kontroll-Apotheken und Interventions-Apotheken geben.

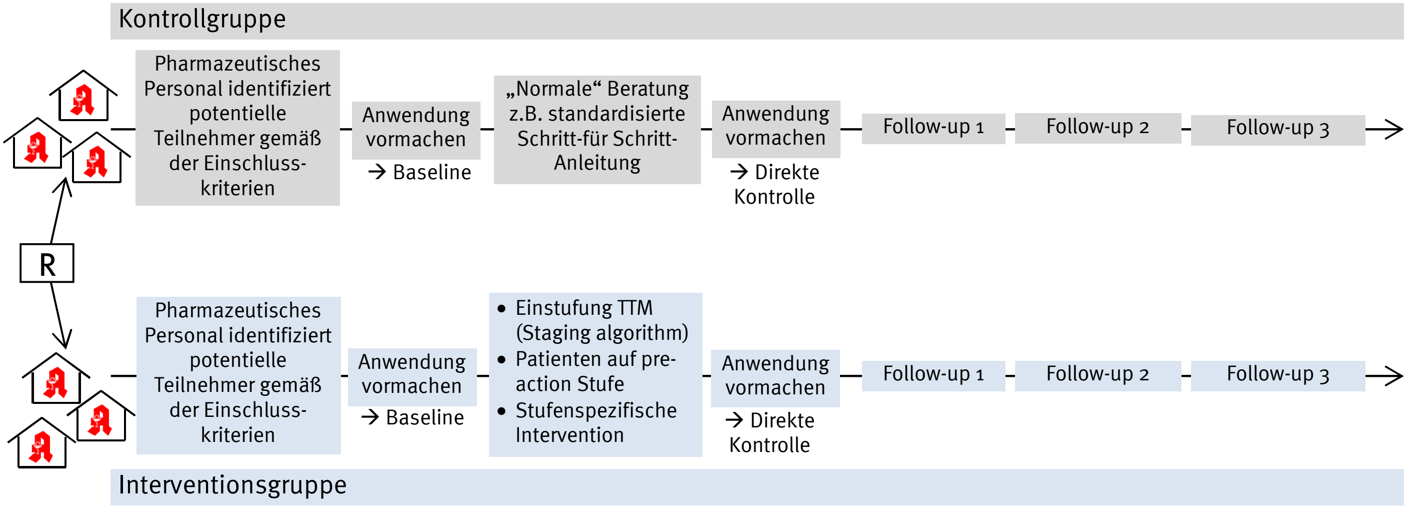

Abbildung 1. Studienablauf. R: Randomisierung, TTM: Transtheoretisches Model

In den Apotheken werden durch das pharmazeutische Personal vor Ort potentielle Studienteilnehmer gemäß den Einschlusskriterien angesprochen (Abb. 1). Es ist möglich im Rahmen dieser Studie die Anwendung unterschiedlicher Darreichungsformen bei demselben Patienten oder pflegenden Angehörigen zu schulen, unabhängig davon wann eine zweite, nach den Einschlusskriterien definierte, Darreichungsform hinzu kommt. Um eine potentiell fehlerhafte Anwendung zu identifizieren, lädt das pharmazeutische Personal den Patienten oder pflegenden Angehörigen ein, die Anwendung seines Arzneimittels vorzumachen (Dauer ca. 2-5

Minuten). Eine solche Demonstration ist im Rahmen der apothekenüblichen Beratung durchaus üblich. Die Demonstration kann mit dem Arzneimittel des Patienten selbst oder auch mit einem Placebo durchgeführt werden. Das pharmazeutische Personal beurteilt dann anhand einer vorab für eine bestimmte Darreichungsform definierten Checkliste (Beispiel siehe Anlage 1) eine gegebenenfalls fehlerhafte Anwendung. Checklisten und die Placebo-Materialien werden von der Abteilung Klinische Pharmakologie und Pharmakoepidemiologie gestellt. In der Kontrollgruppe erfolgen anschließend eine Standard-Beratung (Dauer ca. 3 Minuten) und die Mitgabe von standardisiertem Informationsmaterial, z.B. schriftliche Schritt-für-Schritt – Anleitung (Beispiel siehe Anlage 2). In der Interventionsgruppe wird anhand des „Staging“-Algorithmus (Abb. 2) die individuelle Stufe des Patienten oder pflegenden Angehörigen gemäß dem Transtheoretischen Modell ermittelt. Die Stufe „Maintenance“, d.h. die dauerhaft richtige Anwendung des Arzneimittels, kann mit diesem Algorithmus nicht erfasst werden. Mithilfe dieses Modells sollen insbesondere die Arzneimittelanwenderidentifiziert werden, die sich trotz fehlerhafter Arzneimittelanwendung nicht des Problems bewusst sind und somit unter Umständen nicht motiviert wären eine Schulung oder ausführliche Beratung in Anspruch zu nehmen.

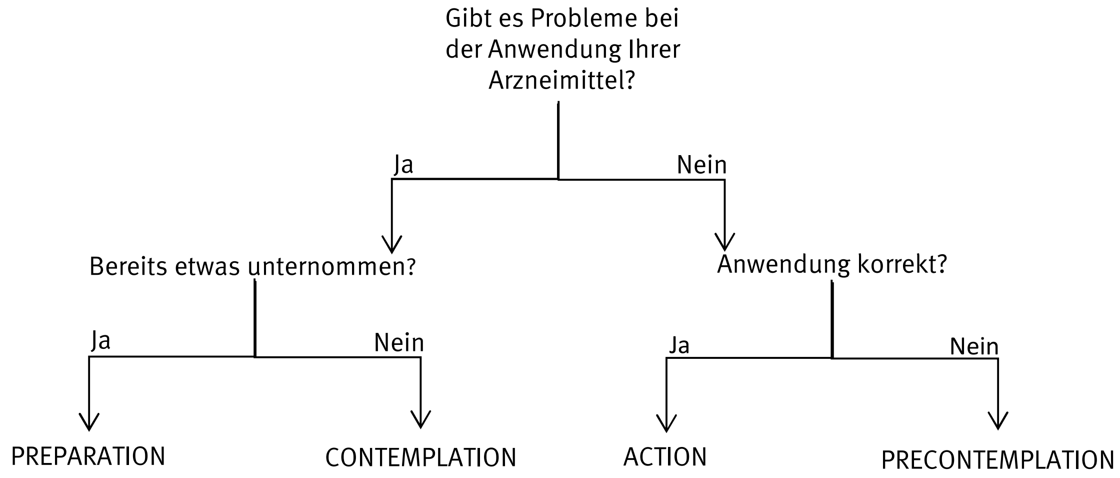

Abbildung 2. „Staging“-Algorithmus

Patienten oder pflegende Angehörige, die sich in einem „Pre-action“ – Stadium befinden werden entsprechend ihrer individuellen Stufe vom pharmazeutischen Personal über die Arzneimittelanwendung informiert und geschult (Tab. 1) (Dauer ca. 5-10 Minuten).

Tabelle 1. Mögliche stufenspezifische Interventionen.

Stage of change	Studienspezifische Definition	Mögliche Intervention	Beispiel
Pre-contemplation	Fehlendes Problem-bewusstsein	z.B. Problembewusstsein schaffen, auf Risiken einer fehlerhaften Anwendung hinweisen	Vorzeigen eines Risikoscores, Information über mögliche Konsequenzen einer fehlerhaften Anwendung und Vorteile einer korrekten Anwendung
Contemplation	Problem erkannt	z.B. auf Risiken einer fehlerhaften Anwendung hinweisen, Möglichkeiten aufzeigen	Vorschlag einer Schulung
Preparation	Problem erkannt, Suche nach Lösungen	z.B. standardisierte Schulungsunterlagen für bestimmte Darreichungsform	Schulung anhand der standardisierten Informationsmaterialien
Action	Arzneimittel wird korrekt angewendet	z.B. regelmäßiges Monitoring	Follow-up-Besuche mit erneutem Vorzeigen der Anwendung, Beurteilung durch das pharmazeutische Personal und ggf. Nachschulung
Maintenance	Arzneimittel wird dauerhaft korrekt angewendet	z.B. regelmäßiges Monitoring	Follow-up-Besuche mit erneutem Vorzeigen der Anwendung und Beurteilung durch das pharmazeutische Personal

Unmittelbar nach der Schulung (Interventions-Gruppe) bzw. nach Ausgabe der standardisierten Anleitung (Kontroll-Gruppe) erfolgt eine erneute Kontrolle der Arzneimittelanwendung mithilfe der Checkliste. Treten weiterhin Fehler auf, erfolgt in der Interventionsgruppe eine entsprechende Nachschulung. In der Kontroll-Gruppe wird der Patient oder pflegende Angehörige mündlich auf die Fehler hingewiesen.

Erst nach dieser, im Rahmen der Routineversorgung üblichen, Beratung, die trotz der Individualisierung nicht deutlich länger dauern sollte, werden die Patienten oder pflegenden Angehörige eingeladen an einer Follow-Up-Erhebung und damit an der Studie teilzunehmen. Die Aufklärung erfolgt durch das pharmazeutische Personal oder einen Mitarbeiter der Abteilung Klinische Pharmakologie und Pharmakoepidemiologie. Möchte der Patient oder pflegende

Angehörige an der Studie teilnehmen und bezeugt dies durch seine schriftliche Einwilligung, werden seine bis dahin anonymisiert erhobenen Daten pseudonymisiert. Nach Einschluss in die Studie werden die Patienten und pflegenden Angehörige gebeten, einen soziodemographischen Fragebogen zu beantworten (Dauer ca. 5 Minuten) (vorläufige Version des Fragebogens siehe Anlage 3). Zusätzlich können z.B. Fingerkraft und kognitive Fähigkeiten als Einflussfaktoren auf den Schulungserfolg erfasst werden. Die weitere Datenerhebung während der Follow-up-Besuche verläuft pseudonymisiert. Es sind drei Follow-up-Besuche nach ca. 1 Monat, 6 Monaten und 12 Monaten geplant. Die Patienten oder pflegende Angehörige werden entweder in der Apotheke zum entsprechenden Zeitpunkt auf das Follow-up angesprochen oder durch Mitarbeiter der öffentlichen Apotheken oder Mitarbeiter der Abteilung Klinische Pharmakologie und Pharmakoepidemiologie rechtzeitig für die Follow-up-Besuche kontaktiert und in die entsprechende Apotheke eingeladen. Die bevorzugte Art der Kontaktaufnahme, z.B. Anruf, Email oder Brief, können die Patienten oder pflegenden Angehörigen bei Einschluss in die Studie wählen (Beispiel siehe Anlage 4). Bei den Follow-ups wird der Patient oder der pflegende Angehörige aufgefordert, die Anwendung erneut vorzumachen, die durch das pharmazeutische Personal oder einen Mitarbeiter der Abteilung Klinische Pharmakologie und Pharmakoepidemiologie anhand der Checklisten evaluiert wird. Bei fehlerhafter Anwendung findet wiederum eine Nachschulung statt. Während des Follow-ups werden die Patienten und pflegenden Angehörige von Mitarbeitern der Apotheke oder der Abteilung Klinische Pharmakologie und Pharmakoepidemiologie kontaktiert, z.B. telefonisch oder schriftlich per Email oder Post, um die Zufriedenheit der Teilnehmer in Bezug auf die Arzneimittelanwendung zu erheben.

Der Studienablauf wird vor Studienbeginn in einer Pilotphase hinsichtlich der Durchführbarkeit durch Studienmitarbeiter und pharmazeutisches Personal evaluiert und evtl. angepasst.

12. Begleittherapie

Entfällt.

13. Sicherheitslabor

Entfällt.

14. Abbruchkriterien

Individuell

Die Zustimmung des Studienteilnehmers (Patienten, pflegende Angehörige und pharmazeutisches Personal) kann jederzeit, ohne Angaben von Gründen und ohne Nachteile, zurückgezogen werden.

Gesamte Studie

Die Studie wird abgebrochen, wenn sich wider Erwarten herausstellen sollte, dass eine potentielle Gefährdung für Studienteilnehmer vorliegen sollte.

15. Statistisches Verfahren

Statistisches Design

Die Fehlerraten der Interventionsgruppe werden mit den Fehlerraten in der Kontrollgruppe mithilfe eines Chi-Quadrat-Tests verglichen. Die Fehlerrate bei der Arzneimittelanwendung vor der Intervention wird mit der Fehlerrate nach der Schulung mithilfe eines McNemar-Tests verglichen. Ein p-Wert $\leq 0{,}05$ wird als statistisch signifikant angesehen. Die Integrierbarkeit der Schulungsmaßnahme in den Apothekenalltag und die Zufriedenheit der Patienten und pflegenden Angehörigen wird qualitativ ausgewertet. Der Einfluss von Kovariablen wie z.B. Darreichungsform, Patientencharakteristika, schulender Person und Anzahl der Arzneimittel auf den Schulungserfolg wird mithilfe einer logistischen Regression ermittelt.

Fallzahlberechnung basierend auf dem Hauptzielkriterium

Aufgrund der Ergebnisse einer randomisierten Studie, die eine aufwendige Schulungsintervention zur richtigen Anwendung von Inhalatoren mit einer Standardberatung verglichen hat, nehmen wir einen Unterschied im Erfolg der Maßnahmen in Kontroll- und Interventionsgruppe von 20% an bei Erfolgraten von $p_1=32\%$ und $p_2=52\%$ [22]. Um einen signifikanten Unterschied zwischen Kontroll- und Interventionsgruppe mittels Chi-Quadrat-Test zu ermitteln (unverbundene Stichproben, $\alpha=0{,}05$; $\beta=0{,}8$) benötigt man, unter Berücksichtigung einer Drop-Out Rate von 15% nach 6 Monaten, einer Clustergröße von 5 Apotheken pro Gruppe und einem Clustereffekt (IntraclusterCorrelation Coefficient, iCC) von 0,02, n=122

Arzneimittelanwendungen einer Darreichungsform z.B. Augentropfen oder Transdermale Therapeutische Systeme pro Gruppe. Bei einer Clustergröße von 10 Apotheken pro Gruppe erhöht sich die Fallzahl pro Darreichungsform z.B. Augentropfen oder Transdermale Therapeutische Systeme auf n=133 pro Gruppe. Die Fallzahlberechnung erfolgt auf der Ebene der Darreichungsformen, weil somit Patienten, die verschiedene Darreichungsformen anwenden, zu ihren einzelnen Arzneimitteln geschult werden können. Das Follow-up nach 12 Monaten wird deskriptiv beschrieben.

16. Ethische und rechtliche Aspekte

Die Untersuchung wird unter Beachtung der in der aktuellen Fassung der Deklaration von Helsinki des Weltärztebundes niedergelegten ethischen Grundsätze für die medizinische Forschung am Menschen durchgeführt.

Das Studienprotokoll wird vor Studienbeginn der zuständigen Ethikkommission der Medizinischen Fakultät Heidelberg zur Bewertung vorgelegt. Es wird nicht mit dem Einschluss von Studienteilnehmern begonnen, bevor nicht das schriftliche, zustimmende Votum dieser Ethikkommission vorliegt.

Bis zum Zeitpunkt des Einschlusses der Patienten oder pflegenden Angehörigen für das Follow-up dient die Studie zur Qualitätssicherung der Beratungsqualität des pharmazeutischen Personals in den teilnehmenden öffentlichen Apotheken. Die Demonstration der Anwendung durch den Arzneimittelanwender und die daran anschließende Beratung durch den Apotheker kann bereits heute in der Routineversorgung stattfinden. Das pharmazeutische Personal wird im Rahmen dieses Qualitätssicherungsprojektes den Arbeitsaufwand, z.B. zusätzlichen Zeitaufwand oder Unterbrechung des Arbeitsablaufes, dokumentieren, um die Alltagstauglichkeit der Schulungsmaßnahme zu evaluieren. Willigen Patienten oder pflegende Angehörige nicht zur Teilnahme in der Studie ein, können die im Rahmen der Beratung erhobenen Daten der Patienten oder pflegenden Angehörigen zur Evaluierung der Beratungsqualität verwendet werden, wenn die Patienten oder pflegenden Angehörigen dies ausdrücklich durch ihre Unterschrift bestätigen. Eine Pseudonymisierung findet nicht statt, da kein Follow-Up dieser Patienten oder pflegenden Angehörigen erfolgt.

Die Teilnahme aller Studienteilnehmer ist freiwillig.

Das pharmazeutische Personal der teilnehmenden Apotheken wird vor Studienbeginn schriftlich und mündlich über Wesen und Tragweite der geplanten Intervention, insbesondere über den möglichen Nutzen und eventuelle Risiken oder Belastungen, die Freiwilligkeit der Teilnahme und über die notwendige Dokumentation ihrer Zustimmung durch Unterschrift auf der Einwilligungserklärung aufgeklärt. Um die geschulten Arzneimittelanwender nicht nur nach Apotheke sondern auch nach schulender Person clustern zu können, erhalten die Apothekenmitarbeiter ein Pseudonym, das auf ihren Berufsstand (z.B. Apotheker oder pharmazeutisch technischer Angestellter) und ihre Berufserfahrung (<1 Jahr, 1-5 Jahre >5 Jahre) hinweist (Beispiel siehe Anlage 5).
Die Patienten und pflegenden Angehörigen werden nach der Schulung schriftlich und mündlich über Wesen und Tragweite der geplanten Follow-ups, über den möglichen Nutzen und eventuelle Risiken oder Belastungen, die Freiwilligkeit ihrer Teilnahme und über die Dokumentation ihrer Zustimmung durch Unterschrift auf der Einwilligungserklärung aufgeklärt.

Die Informationsschrift informiert die Studienteilnehmer (Patienten, pflegende Angehörige und pharmazeutisches Personal) über die Datenerhebung, die in pseudonymisierter Form erfolgt. Des Weiteren werden die Patienten und pflegenden Angehörigen darüber informiert, dass die bis zum Einschluss in die Studie erhobenen Daten, beim Einschluss in die Studie pseudonymisiert werden, um eine Zuordnung der Follow-ups zu ermöglichen. Das Pharmazeutische Personal kann die entsprechende Patienten-Identifikationsnummer nur dann auf die bis dahin erhobenen Daten übertragen, wenn der Patient direkt im Anschluss an die durchgeführte Beratung und Datenerhebung zur Teilnahme an den Follow-up-Besuchen einwilligt. Ist der Patient dazu nicht in der Lage, wird er nicht in die Studie eingeschlossen. Zum Zeitpunkt einer wissenschaftlichen Veröffentlichung sind die Daten für Mitarbeiter der Abteilung Klinische Pharmakologie und Pharmakoepidemiologie pseudonymisiert, für Dritte sind die Daten zu jedem Zeitpunkt faktisch anonymisiert. Die Zustimmung des Studienteilnehmers kann jederzeit, ohne Angaben von Gründen und ohne Nachteile für die weitere medizinische Versorgung, zurückgezogen werden. Wird die Einwilligung zurückgezogen, wird zusammen mit dem Studienteilnehmer entschieden ob dieser mit der Auswertung des bis dahin gewonnenen Datenmaterials einverstanden ist;

anderenfalls werden seine bis dahin erhobenen Daten gelöscht. Wenn einzelne an der Studie teilnehmende Mitarbeiter einer Apotheke ihr Einverständnis zurückziehen, kann die Studie in der Apotheke weitergeführt werden mit dem weiterhin teilnehmenden pharmazeutischen Personal.

Die Namen der Studienteilnehmer und alle anderen vertraulichen Informationen unterliegen der Schweigepflicht nach Strafgesetzbuch (StGB), den berufsrechtlichen Schweigepflichten und den Bestimmungen des Bundesdatenschutzgesetzes (BDSG). Für die Auswertung werden die Daten (z.B. Namen und andere personenbezogene Angaben) pseudonymisiert. Eine Weitergabe von Teilnehmerdaten erfolgt ggf. nur in pseudonymisierter Form. Dritte erhalten keinen Einblick in Studienunterlagen, die nicht-anonymisierte persönliche Daten enthalten. Die Ergebnisse werden in zusammengefasster Form veröffentlicht, ohne Angabe von Namen oder sonstigen persönlichen Daten der Teilnehmer. Die Dateien und Dokumentationsunterlagen werden auf dem zentralen, geschützten Server des Universitätsklinikums archiviert.

17. Unterschriften

Heidelberg, den 05.12.2014

Dr. sc. hum. Hanna M. Seidling

Heidelberg, den 05.12.2014

Prof. Dr. med. Walter E. Haefeli

Heidelberg, den 05.12.2014

Anette Lampert, Apothekerin

18. Referenzen

1. Keers RN, Williams SD, Cooke J, Ashcroft DM. Prevalence and nature of medication administration errors in health care settings: a systematic review of direct observational evidence. Ann Pharmacother 2013;47:237-56.
2. Berdot S, Gillaizeau F, Caruba T, Prognon P, Durieux P, Sabatier B. Drug administration errors in hospital inpatients: a systematic review. PLoS One 2013;8:e68856.
3. Leape LL, Bates DW, Cullen DJ, Cooper J, Demonaco HJ, Gallivan T, Hallisey R, Ives J, Laird N, Laffel G. Systems analysis of adverse drug events. ADE Prevention Study Group. JAMA 1995;274:35-43.
4. Lampert A, Seiberth J, Haefeli WE, Seidling HM. A systematic review of medication administration errors with transdermal patches. Expert Opin Drug Saf 2014;13:1101-14.
5. Hansen B, Matytsina I. Insulin administration: selecting the appropriate needle and individualizing the injection technique. Expert Opin Drug Deliv 2011;8:1395-406.
6. Chowdhury TA, Escudier V. Poor glycaemic control caused by insulin induced lipohypertrophy. BMJ 2003;327:383-4.
7. Sleath B, Blalock S, Covert D, Stone JL, Skinner AC, Muir K, Robin AL. The relationship between glaucoma medication adherence, eye drop technique, and visual field defect severity. Ophthalmology 2011;118:2398-402.
8. Kale A, Keohane CA, Maviglia S, Gandhi TK, Poon EG. Adverse drug events caused by serious medication administration errors. BMJ Qual Saf 2012;21:933-8.
9. Walsh KE, Roblin DW, Weingart SN, Houlahan KE, Degar B, Billett A, Keuker C, Biggins C, Li J, Wasilewski K, Mazor KM. Medication errors in the home: a multisite study of children with cancer. Pediatrics 2013;131:e1405-14.
10. Mager DR. x
11. Kripalani S, Roumie CL, Dalal AK, Cawthon C, Businger A, Eden SK, Shintani A, Sponsler KC, Harris LJ, Theobald C, Huang RL, Scheurer D, Hunt S, Jacobson TA, Rask KJ, Vaccarino V, Gandhi TK, Bates DW, Williams MV, Schnipper JL, PILL-CVD (Pharmacist Intervention for Low Literacy in Cardiovascular Disease) Study Group. Effect of a pharmacist intervention on clinically important medication errors after hospital discharge: a randomized trial. Ann Intern Med 2012;157:1-10.
12. Winfield AJ, Jessiman D, Williams A, Esakowitz L. A study of the causes of non-compliance by patients prescribed eyedrops. Br J Ophthalmol. 1990;74:477-80.
13. Ryan R, Santesso N, Hill S, Lowe D, Kaufman C, Grimshaw J. Consumer-oriented interventions for evidence-based prescribing and medicines use: an overview of systematic reviews. 2011:CD007768.
14. Bertsche T, Bertsche A, Krieg EM, Kunz N, Bergmann K, Hanke G, Hoppe-Tichy T, Ebinger F, Haefeli WE. Prospective pilot intervention study to prevent medication errors in drugs administered to children by mouth or gastric tube: a programme for nurses, physicians and parents. Qual Saf Health Care 2010;19:e26.
15. Sullivan MM, O'Brien CR, Gitelman SE, Shapiro SE, Rushakoff RJ. Impact of an interactive online nursing educational module on insulin errors in hospitalized pediatric patients. Diabetes care. 2010;33:1744-6.
16. Friedman AJ, Cosby R, Boyko S, Hatton-Bauer J, Turnbull G. Effective teaching strategies and methods of delivery for patient education: a systematic review and practice guideline recommendations. J Cancer Educ 2011;26:12-21.
17. Brounéus F, Macleod G, Maclennan K, Parkin L, Paul C. Drug safety awareness in New Zealand: public knowledge and preferred sources for information. J Prim Health Care 2012;4:288-93.
18. Stone JL, Robin AL, Novack GD, Covert DW, Cagle GD. An objective evaluation of eyedrop instillation in patients with glaucoma. Arch Ophthalmol 2009;127:732-6.
19. Souza ML, Meneghini AC, Ferraz E, Vianna EO, Borges MC. Knowledge of and technique for using inhalation devices among asthma patients and COPD patients. J Bras Pneumol 2009;35:824-31.
20. Prochaska JO, Velicier WF. The transtheoretical model of health behavior change. Am J Health Promot 1997;12:38-48.
21. Ficke DL, Farris KB. Use of the transtheoretical model in the medication use process. Ann Pharmacother 2005;39:1325-30.
22. Press VG, Arora VM, Shah LM, Lewis SL, Charbeneau J, Naureckas ET, Krishnan JA. Teaching the use of respiratory inhalers to hospitalized patients with asthma or COPD: a randomized trial. J Gen Intern Med 2012;27:1317-25.
